# Brick by Brick the Wall Is Being Built: Particle-Based Scaffolds for Regenerative Medicine

**DOI:** 10.3390/polym17233227

**Published:** 2025-12-04

**Authors:** Viktor Korzhikov-Vlakh, Lei Wang, Sofia Morozova, Ekaterina Sinitsyna, Tatiana Tennikova, Evgenia Korzhikova-Vlakh

**Affiliations:** 1 Institute of Chemistry, Saint-Petersburg State University, Peterhof, 198504 St. Petersburg, Russia; kat_sinitsyna@mail.ru (E.S.); tennikova@mail.ru (T.T.); e.korzhikova-vlakh@spbu.ru (E.K.-V.); 2 State Key Laboratory of Advanced Inorganic Fibers and Composites, School of Chemistry and Chemical Engineering, Harbin Institute of Technology, Harbin 150001, China; leiwang_chem@hit.edu.cn; 3 Moscow Center for Advanced Studies, 123592 Moscow, Russia; sofiionova@yandex.ru

**Keywords:** particles, 3D-scaffolds, scaffold fabrication techniques, regenerative medicine, tissue engineering

## Abstract

Tissue engineering offers a promising solution by developing scaffolds that mimic the extracellular matrix and guide cellular growth and differentiation. Recent evidence suggests that scaffolds must provide not only biocompatibility and appropriate mechanical properties, but also the structural complexity and heterogeneity characteristic of natural tissues. Particle-based scaffolds represent an emerging paradigm in regenerative medicine, wherein micro- and nanoparticles serve as primary building blocks rather than minor additives. This approach offers exceptional control over scaffold properties through precise selection and combination of particles with varying composition, size, rigidity, and surface characteristics. The presented review examines the fundamental principles, fabrication methods, and properties of particle-based scaffolds. It discusses how interparticle connectivity is achieved through techniques such as selective laser sintering, colloidal gel formation, and chemical cross-linking, while scaffold architecture is controlled via molding, templating, cryogelation, electrospinning, and 3D printing. The resulting materials exhibit tunable mechanical properties ranging from soft injectable gels to rigid load-bearing structures, with highly interconnected porosity that is essential for cell infiltration and vascularization. Importantly, particle-based scaffolds enable sophisticated pharmacological functionality through controlled delivery of growth factors, drugs, and bioactive molecules, while their modular nature facilitates the creation of spatial gradients mimicking native tissue complexity. Overall, the versatility of particle-based approaches positions them as prospective tools for tissue engineering applications spanning bone, cartilage, and soft tissue regeneration, offering solutions that integrate structural support with biological instruction and therapeutic delivery on a single platform.

## 1. Introduction

Despite the ongoing development of medicine, the loss or damage of tissue structure and function caused by trauma, aging, tumoral removal, or infectious disease represents a huge amount of medical cases [1,2,3,4]. This problem is negatively affected by the tissue aging associated with growing life expectancy [5]. Currently applied clinical practices, such as autografts, allografts, and implants, have their definite limitations [6]. The first ones can only be taken from a limited number of locations within the human organism, and the volume of tissues that can be taken without negative consequences is also highly restricted [7]. Allografts are also prone to foreign body responses. Implants can be designed for a much broader range of cases, but still cannot provide lost tissue remodeling and vascular ingrowth, which often leads to the malfunction of surrounding tissues [8]. In light of the foregoing, the development of innovative solutions that can improve the healthcare of the aging population and those living with disease continues to be a global challenge.

Tissue engineering represents a promising interdisciplinary scientific field that offers a unique opportunity to grow living tissue in vivo or in vitro in order to replace failing or malfunctioning tissues in a patient by using the patient’s own cells [8,9]. This field has been defined by Laurencin [10] as “the application of biological, chemical and engineering principles toward the repair, restoration or regeneration of living tissues using biomaterials, cells and factors alone or in combination”. The goal of tissue engineering can be reached by the development of precisely constructed media for supporting cells and guiding their growth. These media are called scaffolds, and they take over the role of the natural extracellular matrix (ECM). The ECM provides cells with a supportive framework of structural proteins, carbohydrates, and signaling molecules [6]. An ideal scaffold must mimic the ECM and be composed of a biomaterial that provides all the necessary signals for cells to grow, differentiate, and interact, forming the target tissue structure [11].

The design of scaffolds plays a critical role in the success of regenerative medicine [11]. Their primary function is to guide the growth of cells, either those seeded within their porous network in vitro or those infiltrating from host tissue in vivo. Since most mammalian cells are anchorage-dependent, scaffolds must first provide a suitable substrate for attachment [9]. With many tissue engineering strategies now relying on stem cells [12,13,14,15], scaffolds must additionally support their differentiation into specific cells [8]. Other essential requirements include biocompatibility and biodegradability to enable tissue development and remodeling, as well as ease of processing into desired shapes. A highly porous architecture is crucial for efficient nutrient delivery and waste removal. In the context of connective tissue regeneration, scaffolds must also possess sufficient mechanical strength and dimensional stability [11,16,17].

Over the past decade, it has become increasingly evident that, in addition to the features mentioned above, scaffolds must also replicate the complexity and heterogeneity of the natural ECM [18,19,20,21]. It is well known that the natural ECM contains gradients of special signals and structural heterogeneity, which enable directed cell migration, vascularization, and the regulation of tissue density [11]. Thus, the development of methods for fabricating scaffolds with gradients of mechanical and biological properties is currently one of the crucial tasks for the success of tissue engineering.

One of the strategies for regulating the mechanical and biological properties of scaffolds is to incorporate micro- and nanoparticles into their structure [22]. For decades, such particles have been extensively studied for drug and gene delivery, progressing from the delivery of small molecules [23,24,25] to complex biomolecules like proteins [26,27,28] and nucleic acids [29]. These particles can be fabricated from a wide range of materials, including polymers, ceramics, and metals [30]. Numerous approaches have employed them as a minor phase within composite scaffolds to enhance material properties [31,32]. For example, silver nanoparticles have great significance in antimicrobial applications due to their potent bactericidal activity [33]. Ceramic particles, especially calcium phosphate [34], hydroxyapatite (HA) [35], as well as bioglass [36,37] and biosilica [37], are of interest because they allow the mechanical strength to be increased and enhance the bioactivity of scaffolds. As an example, biosilica particles isolated from the marine sponge *Axinella infundibuliformis* and incorporated into marine-derived collagen materials promoted fibroblast adhesion and exhibited osteoinductive properties [37]. Polymeric particles with diverse composition and tunable properties have also demonstrated a significant impact on the characteristics of scaffolds [38,39]. A key advantage of these polymeric systems is their capacity for the encapsulation and controlled release of bioactive molecules that are critical for tissue regeneration [40,41,42]. Beyond their role within the scaffold bulk, nanoparticles are also highly valuable as surface-modifying agents. Their application in this context allows control over surface topology, which is crucial for cell–material interactions [19,43,44]. Despite considerable research efforts, the creation of scaffolds with a controlled microstructure that accurately mimics the intercellular matrix of native tissue still represents a major challenge. In this context, the application of particles to fine-tune the bulk, surface, pharmacological, and biosignaling properties of scaffolds is of great interest [22,45,46,47].

As previously discussed, particles are commonly incorporated as a minor phase in numerous composites and serve as reinforcing agents or drug delivery vehicles. In contrast, scaffolds where particles themselves constitute the primary matrix-forming material are far less common. At the same time, particle-based scaffolds expand the poorly explored niche of materials that can provide very good performance as ECM-mimicking materials. Fundamentally, the precise combination of different particles with various natures, rigidities, sizes, surface modifications, etc., can serve as a versatile “toolbox” to precisely control the mechanical, biological, and pharmacological properties of scaffolds. Organizing multiple particle types into a material is a promising approach for developing well-vascularized scaffolds that effectively support cell ingrowth and new tissue formation.

**Figure 1:**
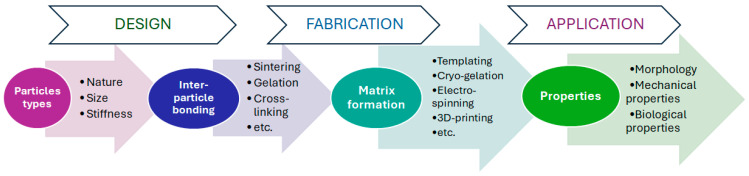
The conceptual framework of the review.

Given the significant potential of particle-based scaffolds, this review focuses on structures where particles constitute the primary matrix-forming phase, or where materials are enriched in particles rather than acting as a minor additive. To contextualize this review, it is important to acknowledge several excellent recent review papers on related topics. Xuan L. and colleagues have comprehensively reviewed microgel systems used in tissue engineering [48], while Bektas and colleagues have discussed in detail the use of microgels for bone tissue regeneration [49]. However, these reviews focus specifically on microgels as functional units rather than on their use as building blocks for scaffold formation. Similarly, a review on microspheres for bone and cartilage tissue engineering [50] provides extensive information on fabrication methods but does not detail the critical aspect of forming interparticle bonds. This specific topic is covered by other notable reviews on granular hydrogels [51,52]. While these reviews are foundational, they are largely confined to microgel-based systems and do not consider other types of particles, such as polyester-based, inorganic ones, etc. Thus, this review aims to fill this gap by providing a comprehensive analysis of the vast range of particles used in scaffold construction. The framework of the review is illustrated in Figure 1.

## 2. Types of Particles for Preparation of Particle-Based Scaffolds

**Figure 2:**
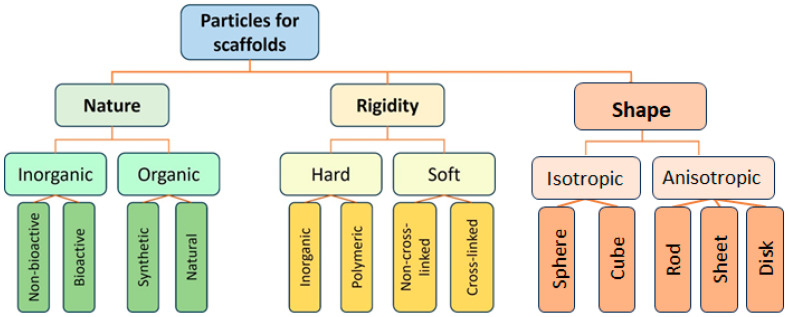
Classification of particle types used for scaffold preparation.

Different types of particles can serve as building blocks for scaffold construction. Just as the quality of bricks determines the characteristics of a building, the choice of particles is crucial, as it predetermines the material’s final properties (Figure 2). These particles act as the foundational units, and their selection directly dictates the scaffold’s physicochemical properties, morphology, and biological performance.

**Figure 3:**
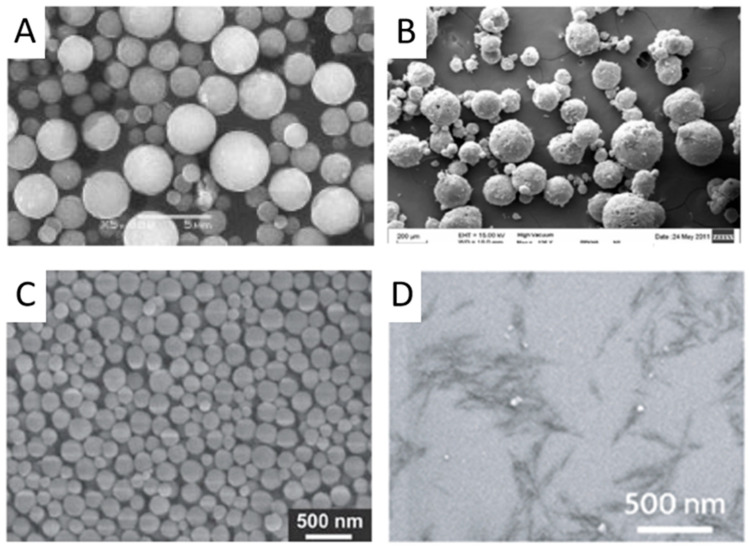
SEM images of some inorganic and polymer particles used for scaffold preparation: (**A**) hydroxyapatite particles (HA). Reproduced with permission from [61] Copyright© 2008, John Wiley and Sons. (**B**) poly(D,L-lactic acid) (PLA) particles coated with CTAB. Reproduced with permission from [56] Copyright© 2014, American Chemical Society. (**C**) gelatin A particles. Reproduced with permission from [57] Copyright© 2011, John Wiley and Sons. (**D**) cellulose nanocrystals (CNC). Reproduced with permission from [58] Copyright© 2023, Elsevier.

Particles used in scaffold fabrication can be classified by their nature into inorganic and organic ones. Historically, the first particles applied to scaffolds were inorganic [53], which was dictated by the type of process used for the formation of such scaffolds, namely sintering. However, these early scaffolds were primarily suitable for bone tissue regeneration, while the repair of other tissues requires more versatile materials [54]. This motivated researchers to explore polymers and polymer particles as scaffolding materials. Polymer particles can be further subdivided into those based on synthetic and natural polymers. Synthetic polymers usually offer greater control over the mechanical properties of the material and its microstructure, whereas natural polymers allow the formation of biomimetic and cell-instructive structures. A valuable and widely used property of polymeric particles is their suitability for drug encapsulation and sustained release. Consequently, they can function simultaneously as building blocks for the scaffold and as delivery vehicles for bioactive substances or drugs, enhancing the scaffold’s performance both in vitro and in vivo. Particles can also be classified by their shape into isotropic (sphere or cube) or anisotropic (disk, sheet, rod, whisker, and other). The shape of the particles directly affects the scaffold’s pore size and geometry. To date, the majority of particles used have an isotropic spherical shape and form a sphere-packaging type of microporous structure (Figure 3) [55,56,57]. The anisotropy of particles dramatically influences pore size and shape. For example, rod-shaped cellulose nanocrystals (CNC) form gels with a fibrillar macroporous structure [58,59,60]. SEM images of some inorganic and polymer particles are shown in Figure 3.

Particles for scaffold fabrication can also be classified by their rigidity into hard and soft particles [62]. Inorganic particles are generally hard and, as previously mentioned, are more suitable for regenerating hard connective tissues. They have been widely used in various composites, predominantly to enhance the mechanical properties of the scaffolds. Furthermore, particles based on hydroxyapatite (HA) and tricalcium phosphate (TCP) are known to induce osteointegration of scaffolds in vivo. In contrast, soft nanoparticles have been successfully applied for the regeneration of soft tissues, such as the skin [63] and liver [64].

Recent studies have demonstrated that soft polymer microgels based on functionalized poly(ethylene glycol) (PEG) [65], gelatin methacrylate [66], hyaluronic acid [67], poly(N-vinyl caprolactam) [68], and other polymers show promise for producing microporous granular scaffolds and colloidal gels. Notably, the stiffness of these granular microparticles can be tailored by selecting polymers with different chain flexibilities, adjusting the polymer molecular weight, or modifying the degree of crosslinking within the particles [52].

Modern trends in granular scaffold design include the creation of complex multifunctional compositions. For example, Rui-Chian Tang et al. developed crescent-shaped particles consisting of 4-armed PEG vinyl sulfone as a polymer backbone. These particles incorporated several functional components: peptides (K-peptide, Q-peptide, and RGD peptide) to promote cell adhesion, an MMP-sensitive cross-linker, gelatin as a sacrificial material, and Alpha Fluor 488 for visualization. These hydrogel microparticles have an average diameter of 50 μm with internal cavity sizes ranging from 68 to 84% of the particle diameter after equilibration. The particles were covalently annealed via Michael addition chemistry, where dithiol-modified PEG reacted with residual vinyl sulfone groups on the particle surface. These granular hydrogels were fabricated using a microfluidic water-in-oil droplet emulsion technique that also included a two-phase separation of the PEG and gelatin. This process was followed by overnight incubation at 37 °C, emulsion breaking, and ethanol sterilization [69].

Table 1 summarizes the recent studies on the types of particles and methods for fabricating particle-based scaffolds. It is noteworthy that in many studies, scaffolds have been formed by combining both inorganic and organic particles into interparticle composite structures. This interparticle approach allows for a high degree of control over the scaffold’s mechanical properties.

## 3. Peculiarities of Particle-Based Scaffold Preparation

Modern tissue engineering requires scaffolds to be supermacroporous supports with interconnected pores and mechanical properties that mimic those of the native tissue. Therefore, fabricating a particle-based scaffold involves organizing particles into a three-dimensional macroporous material with satisfactory mechanical stability. Extending the brick analogy from the title, the “bricks” (particles) must bond to one another through specific interactions. This process primarily addresses two key challenges: (1) ensuring particle connectivity to form the continuous network; and (2) defining the resulting 3D structure and pore architecture. Particle interconnectivity can be achieved through either non-covalent cohesion, which relies on attractive physical forces, or covalent bonding, which involves the formation of chemical bonds between particles. Meanwhile, pore formation can be realized through various methods, such as colloidal gel assembly, cryogelation (freeze-drying), porogen leaching, or 3D printing.

### 3.1. Interparticle Network Formation

#### 3.1.1. Particle Sintering and Fusion

**Figure 4:**
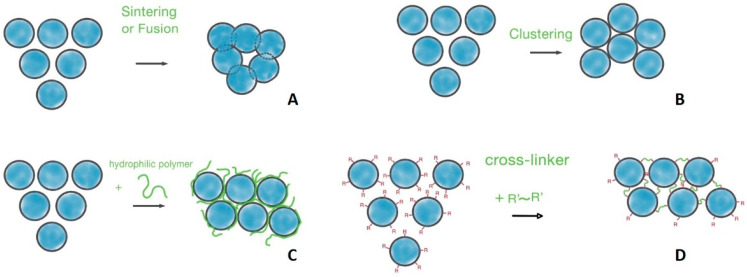
Principal methods of particle interconnection: (**A**) sintering or fusion; (**B**) clustering and aggregation of particles; (**C**) hydrophilic polymer-mediated interaction; (**D**) covalent cross-linking.

Sintering powder particles into a unified matrix was historically one of the first methods used for scaffold preparation. This technique is based on the fusion of particles through the application of heat or pressure (Figure 4A). It is important to note that this process does not involve melting the particles into a liquid phase. Instead, material diffusion across the boundaries of the particles fuses them into a continuous material structure. Sintering can be used for both inorganic (e.g., bioceramic) and polymeric particles.

**Figure 5:**
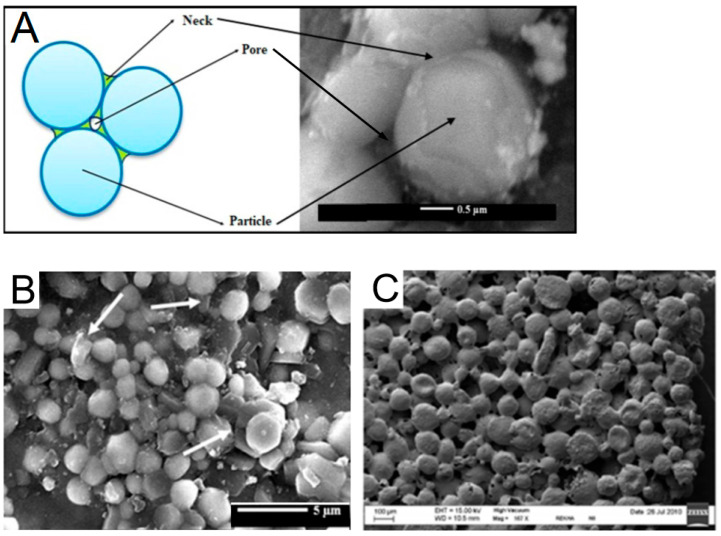
SEM images showing cross-section morphology of matrices obtained by particle sintering or fusion: (**A**) Scheme of sintering and a fragment of the sintered scaffold. Reproduced with permission from [84] Copyright© 2019, John Wiley and Sons. (**B**) A two-step sintered HA-Y_2_O_3_ biocomposite. Reproduced with permission from [84] Copyright© 2019, John Wiley and Sons. (**C**) PDLLA-CTAB membranes after fusion of particles by ethanol. Reproduced with permission from [56,57] Copyright© 2014, American Chemical Society.

One of the most advanced techniques for the sintering of particles is selective laser sintering (SLS), which is based on the application of laser beam power to heat and fuse the particles. The SLS process offers significant benefits over other techniques because of its ability to produce parts with high dimensional accuracy, excellent mechanical properties [80], and good surface quality [81]. In particular, the SLS technology can accurately control the size, shape, and connectivity of bone scaffolds. Schematical representation of sintering model geometry of spherical particles as well as the SEM images of materials based on sintered inorganic and polymer particles are shown in Figure 5.

Sintering was first applied to fabricate scaffolds from bioceramic particles, such as bio-derived calcium phosphate [71], HA [82], bioglass [83], etc. For example, HA particles can be gelled, and the resulting structures sintered, to form a sponge-like ceramic matrix [72]. However, the need to optimize porosity, interpore connectivity, and surface area, as well as to tune mechanical properties, spurred the development of composite scaffolds combining polymeric and inorganic particles. This approach leverages the high elasticity of polymers and the rigidity of inorganic components to produce materials with enhanced and customizable properties via additive manufacturing. For instance, in a recent study, authors successfully used SLS to fabricate a polyamide (PA12) and HA interparticle composite, which demonstrated good mechanical and biological properties [55]. In another study, spherical composite porous particles were produced by incorporating milled bioglass into or onto foamed PLGA spheres [78]. These particles were subsequently laser-sintered into 3D materials exhibiting high porosity and a tunable microstructure.

Polymeric particles, such as PLA coated with hydrophobized chitosan, can also be sintered by surface-selective laser sintering (SSLS) to produce 3D porous materials [77]. It was shown that a hydrophobized chitosan layer effectively stabilized the interface during microparticle fabrication and provided a well-balanced surface hydrophilicity that facilitates water adsorption. During the sintering process, this adsorbed water acted as a sensitizer, responsive to infrared laser irradiation and transferring the heat to the subsurface layer of the PLA particles. This process was optimized to melt only the particle surface successfully, leaving the bulk of the microparticle intact [85].

Polymer particles can be fused not only by heating, but also by swelling the surface layer. For instance, PLA particles can undergo swelling-induced fusion in ethanol [86]. It is known that the stability of aqueous particle suspensions is often maintained by different surfactants. However, adding a non-polar or less polar solvent can disrupt this stability, leading to particle aggregation and the formation of organized 3D structures [87]. This principle was demonstrated in a study where surfactant-coated PLA particles in 100% ethanol fused at room temperature into membrane-type structures [56] (Figure 5C). The authors attributed this fusion to the desorption of the surfactant from the particle surface, accompanied by the formation of polymeric bridges between particles. These bridges represent the transient regions where polymer molecules were solubilized by the ethanol, enabling the formation of a continuous membrane-type structure.

While particle sintering is a powerful and well-established method for creating strong, porous scaffolds from durable materials such as titanium and hydroxyapatite, it has significant limitations for advanced tissue engineering. These include the following: (1) high-temperature or solvent-mediated processing; (2) the formation of closed pores; (3) non-uniform shrinkage; (4) challenges with multi-material and composite scaffolds preparation; and (5) difficulties in incorporating bioactive molecules and living cells. Taking into account such limitations, particle-based scaffold preparation technology has shifted toward low-temperature and room-temperature annealing strategies, which will be reviewed in the following sections.

#### 3.1.2. Particle Clustering and Aggregation in Colloidal Gels

When particles interconnect via attractive, non-covalent surface interactions rather than interpenetration, the material forms through a process of clustering and subsequent aggregation (Figure 3B). This process is primarily driven by the reduction in the particles’ excess free surface energy and the cohesion between chemical groups on their surfaces.

Various non-covalent forces can cause direct particle attraction, clustering, and further aggregation into materials, which are commonly known as “colloidal gels” [88]. The attraction between particles is generally caused by van der Waals [89], hydrophobic [90], solvation [91], or electrostatic [70] forces. Such interactions between particles result in the formation of a heterogeneous structure characterized by the assembly of colloidal particles into strands, which form a mechanically stable particulate network.

The nature of these interparticle forces, along with the particle type, critically affects the scaffold’s rheological and mechanical properties. For instance, scaffolds assembled through electrostatic attraction between oppositely charged particles [74,76,92] aggregate more rapidly and exhibit greater mechanical stability than those formed by hydrophobic interactions. This makes electrostatically bonded scaffolds particularly suitable for tissue engineering applications.

Various biopolymer-based oppositely charged particles have demonstrated the possibility of forming gels upon mixing [57]. For example, such gels can be obtained using dextran microspheres modified by methacrylic acid (Dex-MA) or dimethylaminoethyl methacrylate (Dex-DMAEMA) [74] and gelatin (Gel) particles from different sources (cationic Gel A from porcine skin and anionic Gel B from bovine skin). A solid content of at least 10–15 wt% is generally required to achieve an elastic modulus above 500 Pa. However, the properties of the resulting gels vary significantly. The Dex-MA/Dex-DMAEMA gel exhibits plastic behavior at a low mechanical stress (~10 Pa), indicating that its electrostatic bonds can be easily broken. At the same time, once the stress is removed, the gel shows self-recovery. Furthermore, this gel stability is highly sensitive to ionic strength. In particular, a sharp drop in storage modulus is observed at ionic strength above 0.5 M. In contrast, mixtures of Gel A and Gel B form substantially more stable structures. At a 20% (*w*/*v*) solid content, they achieve an elastic modulus of approximately 10 kPa and remain stable at a physiological ionic strength that indicates their superior potential as injectable biomedical scaffolds. Moreover, the authors compared gels based on gelatin microspheres and nanospheres and showed that the latter were considerably more elastic. This enhancement was attributed to the larger specific surface area of the nanospheres, which creates a greater interparticle contact area and thus provides higher resistance to shear forces [57].

Methacrylated gelatin (GelMA) nanospheres have recently been structured into 3D supports via colloid gelation. In this process, the GelMA particles are initially bonded by multiple reversible non-covalent bonds. This design confers shear-thinning properties: under the stress of a printer nozzle, the reversible bonds break, allowing the particles to flow like a liquid. At the same time, when the shear stress is removed, the bonds rapidly re-form, restoring the solid-like gel structure and “fixing” the printed filament in place [93].

**Figure 6:**
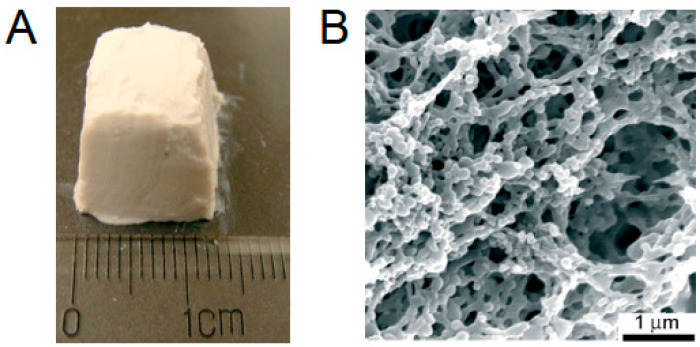
Scaffolds made of PLGA nanoparticles with opposite charges (PLGA-PEMA and PLGA-PVAm): (**A**) photo of stable material made from 20 wt% colloidal gels (1:1 mass ratio). (**B**) SEM image of material obtained at 7:3 mass ratio of PLGA-PEMA/PLGA-PVAm. Reproduced with permission from [76]. Copyright© 2007, John Wiley and Sons.

Wang et al. reported the formation of scaffolds by gelling oppositely charged particles made of synthetic biodegradable aliphatic polyester, namely poly(D,L-lactic-*co*-glycolic acid) (PLGA) [76]. The surface charge was imparted via nanoprecipitation of the PLGA solution into polyvinylamine (PVAm) to create positively charged particles, and into poly(ethylene-*co*-maleic acid) (PEMA) for negatively charged ones. Interestingly, that excess of negatively charged particles resulted in more rigid structures. The authors attributed this to the larger zeta potential of the positively charged nanoparticles, suggesting that an excess of negatively charged particles created a more balanced overall charge. While the resulting structures were generally fluid, a particle concentration of 20% produced relatively stable gels (Figure 6). These gels can be used directly or further stabilized by gel lyophilization [76]. Similar results were obtained when a PLGA particle charge was generated by blending with chitosan (positive) and alginate (negative) for the preparation of oppositely charged particles [92].

The clustering and aggregation of diverse particles with different stiffnesses provide a great opportunity to fabricate organic–inorganic composites with finely tuned mechanical and biological properties [94]. According to Wang et al. [75], nanostructured colloidal composite gels can offer several advantages over conventional bulk composites, including the following: (i) enhanced control over macroscopic scaffold properties by fine-tuning the sub-populations of particulate building blocks [95,96,97]; (ii) injectability/moldability for minimally invasive application into irregular defects [74,76]; (iii) in situ gel formation without the need for potentially cytotoxic gelling/cross-linking agents [95,98]; and (iv) facile incorporation of therapeutic agents [99,100].

It has been shown that calcium phosphate and gelatin nanoparticles can co-assemble into heteroclusters, forming organic–inorganic composites [75]. These systems exhibited superior viscoelastic properties at a gelatin solid content of 10 wt% and a CaP/Gel B ratio between 0.5 and 1. The inclusion of CaP nanoparticles (zeta potential: +3 mV) with the anionic Gel B nanoparticles (zeta potential: −20 mV) significantly enhanced the material’s stiffness. The storage modulus (G’) increased dramatically from 7 to 10 kPa in gels without CaP to as high as 48 kPa in the composite heteroclusters.

Diba et al. developed a smart way to initiate particle aggregation into a material by gradually altering the pH of a colloidal solution [70]. The authors correctly noted that colloidal gels formed by direct mixing often suffer from uncontrollable, nonuniform aggregation and phase separation, which compromise their structural integrity and mechanical strength. To overcome this, they prepared suspensions of gelatin and silica nanoparticles in a basic medium (pH 11), where both components were negatively charged and stable. Glucono-delta-lactone (GDL) was then introduced into the system as a slow acidifier. As GDL hydrolyzed, it gradually lowered pH below the isoelectric point of gelatin, resulting in its charge changing to positive. This triggered the self-assembly of the newly positive gelatin particles with the negative silica particles, allowing the formation of superior gels with a storage modulus (G’) ranging from 10 to 50 kPa.

The interaction between organic and inorganic particles can be further enhanced through surface modification. In a follow-up study, Diba et al. functionalized gelatin particles with calcium-binding bisphosphonate groups, which specifically increased their attraction to calcium-containing bioactive glass particles [101]. The aggregation of these modified particles produced gels that exhibited frequency-independent solid-like behavior. Furthermore, the mechanical properties of these materials could be quite precisely tuned by varying the ratio of bioglass/gelatin nanoparticles.

The reversible nature of non-covalent interparticle interactions is critical for developing advanced biomaterials, enabling the creation of both injectable in situ-forming scaffolds and self-healing systems [70,101]. This self-healing capability was vividly demonstrated in a gelatin/silica nanoparticle gel, which recovered over 100% of its original storage modulus after being subjected to destructive shear [70]. The authors explained this strengthening as a structural reorganization into a more stable configuration. Confocal microscopy confirmed that the recovered gel featured thicker, longer strands and a lower node density compared to the initial network structure. This “strand thickening” effect is likely a result of high shear forces breaking the initial bonds and allowing particles and clusters to reassemble into a more densely packed, robust architecture.

Similarly, a composite gel of calcium phosphate and gelatin nanoparticles also exhibited strong self-healing. It was characterized by almost 70% recovery of initial gel elasticity within 5 min after severe gel network destruction [75]. Together, these findings underscore how reversible bonds facilitate not only injectability but also intrinsic repair mechanisms, making such colloidal gels highly promising for applications requiring durability and resilience.

An interesting approach for the controllable in situ formation of a 3D gel matrix utilizes thermogelable poly(*N*-isopropylacrylamide-*co*-2-hydroxyethyl methacrylate) (P(NIPAAM-*co*-HEMA) microparticles [64]. At low temperatures, the microgel particles were swollen and stabilized by electrostatic repulsion. However, when heated to 37 °C, the microgel particles shrink due to their thermosensitive nature. This shrinkage induces hydrophobic interactions between the particles, leading to the formation of a physically crosslinked 3D network.

This assembly of particles into a bulk material is a hallmark of granular gels, where the fundamental principle is jamming [51]. In these systems, a transition from a liquid-like to a solid-like state occurs based on packing density [52]. When the particle volume fraction (φ) of microgels exceeds 0.58, the system reaches a “random loose packing” state and jams. At φ ≈ 0.64, it achieves a “random close packing”, which often requires microgel deformation. In this jammed state, particles are immobilized by physical constraints and interactions with their neighbors until sufficient stress is applied to overcome the packing force. A key property of these jammed systems is shear-thinning: their viscosity decreases under shear stress, enabling injectability and 3D printing [51,52]. This principle has been demonstrated with microgels based on PEG [102], polyacrylamide [51], poly(N-vinylcaprolactam) [52], gelatin [93], and hyaluronic acid [67].

To ensure the stability of interconnected granular gels, secondary cross-linking between particles is often essential. Physical cross-linking relies on non-covalent interactions such as host–guest interactions, electrostatic interactions, and hydrogen bonding [65,103,104]. These interactions are more spontaneous and dynamically reversible, supporting properties like self-healing and injectability. Covalent cross-linking strategies for these systems will be discussed in a subsequent section.

Colloidal gels represent a significant paradigm shift from traditional monolithic hydrogels toward more modular and porous architectures. However, their primary limitations arise from the fundamental trade-off between establishing a highly porous network and maintaining robust mechanical integrity. While post-assembly strategies like annealing have successfully addressed many of these challenges, they introduce additional procedural complexity.

The field is actively pursuing several innovative paths to overcome these challenges, including the following:

- The development of stronger, more sophisticated, and bio-orthogonal annealing chemistries (e.g., click reactions);
- Advancements in high-throughput and precise microgel fabrication techniques;
- The creation of composite systems that integrate the porosity of colloid gels with reinforcing fibrous networks or a stronger secondary matrix.

Despite the current limitations, the exceptional injectability, high porosity, and modular nature of colloidal gels establish them as a uniquely powerful platform for non-load-bearing tissue regeneration, 3D bioprinting, and drug delivery applications.

#### 3.1.3. Flexible-Chain Polymer-Mediated Interaction

Attractive interactions between particle surfaces may be mediated by dissolved macromolecules that cause their clustering and aggregation (Figure 3C). The resulting architecture depends on several factors: the interaction energy between particles, their volume fraction, and the range of the interaction, which is influenced by molecules in the surrounding medium [105,106,107]. By modulating these parameters, one can steer the process toward distinct outcomes. Fast diffusion-limited clustering occurs when attractions are strong and long-range, producing tenuous branched networks with low fractal dimensions. In contrast, slow reaction-limited cluster aggregation results from weaker, short-range attractions, forming more compact and dense aggregates with higher fractal dimensions [108,109]. For a material at a fixed volume fraction, varying the interaction range is a key control mechanism. Highly attractive interactions produce branched strands, while less attractive ones form compact aggregates [105]. This control can be achieved by introducing ionic low molecular components [110] or specific macromolecules [111] to the medium.

Yuan et al. demonstrated that the microstructure of cationic polyurethane colloidal materials can be controlled by the type of anion mediating their electrostatic aggregation [105]. When aggregated by small anions in phosphate-buffered saline, the particles formed dense aggregates. In contrast, using the carboxylate groups of a poly(acrylic acid) resulted in a tenuous, branched aggregate [105]. The authors used these features to spatially control the localization of cues within the scaffold, thereby enhancing angiogenesis. The discussion of this issue will be presented below.

Flexible-chain polymer-mediated interactions are excellent for creating injectable, self-healing, and stimuli-responsive scaffolds. However, for applications requiring high mechanical strength, predictable long-term stability, and resistance to a dynamic biological environment, the strategy of the covalent annealing of particles provides a more reliable choice.

#### 3.1.4. Particle Cross-Linking

Covalent crosslinking into a unique monolithic structure is preferred for applications requiring greater stability (Figure 4D). This approach provides a versatile route for fabricating scaffolds with controllable properties. It is important to note that covalent cross-linking can be applied after initial particle clustering or can drive the aggregation process itself.

For instance, cross-linking after clustering was applied by Cho et al. [112]. The authors reported the preparation of a thermally responsive hydrogel by first aggregating positively charged microparticles with anionic poly(acrylic acid), followed by covalent cross-linking of particles with glutaraldehyde [112]. This cross-linking formed a stable interparticle network that imparted thermo-sensitivity to the entire matrix and allowed for the incorporation of other functional particles.

In the study by Siders et al., particle hydrogels were used to create microporous scaffolds through annealing hyaluronic acid-based microgel particles [67]. The authors compared several cross-linking techniques, namely, transglutaminase-mediated amide bond formation, thiol-ene Michael addition, and amine/carboxylic acid-based cross-linking. The resulting materials combined microporosity with tunable mechanical properties, making them promising for injectable tissue engineering applications where both cell infiltration and structural integrity are required.

Recently, Morozova et al. reported a method for creating fibrillar hydrogel scaffolds using the Diels–Alder reaction to cross-link two types of cellulose nanocrystals (CNCs) with furan and maleimide groups [113]. The gelation was studied at various component ratios and temperatures ranging from 20 to 60 °C. The authors determined that a 5 wt% CNC concentration was required to form a stable hydrogel with minimal swelling in aqueous media across different pH.

In our study, we have applied UV-initiated cross-linking to assemble scaffolds from poly(lactic acid) (PLA) nanoparticles and CNC [114]. Both PLA nanoparticles and CNC were methacrylated in order to provide reactivity in a free-radical cross-linking reaction. To enhance the material’s properties, we incorporated gelatin–methacrylate as a macromolecular cross-linker, which was shown to produce matrices with significantly improved viscoelasticity.

Another recent study employed a similar approach. GelMA particles were assembled into 3D materials via a colloidal gel approach and then converted into a permanent, robust scaffold by exposing the formed structure to UV light in the presence of a photoinitiator. This process creates a continuous, covalently bonded fibrous network that fuses the particles into a cohesive whole, blurring their individual boundaries [93].

Chemical reaction-driven annealing of hydrogel microparticles represents the popular approach to granular microgels. The process is based on the formation of covalent bonds between adjacent microgels. These reactions often require modifications to functional groups on molecular chains or the surface of microgels. Popular chemical annealing strategies include enzyme-catalyzed reactions [115], free radical polymerization [116], and click reactions [117].

Hung-Pang Lee et al. presented hydrazone bond formation as a key microgel cross-linking strategy. The proposed method involved mixing aldehyde- and hydrazide-functionalized microgels to provide interparticle cross-linking through reversible hydrazone bonds [102]. This strategy imparted shear-thinning and self-healing properties to the resulting materials.

However, several challenges are associated with interparticle cross-linking in scaffold preparation. Firstly, it can compromise the injectability that makes particle-based systems so attractive. Secondly, the cross-linking chemistry itself is often complex. Finally, there is a persistent risk of cytotoxicity from the reagents or reaction conditions, which is a critical concern when cells are present. A thorough understanding of these trade-offs is therefore crucial for selecting an appropriate cross-linking strategy for a given tissue engineering application.

### 3.2. Scaffold Shape Control and Pore Structure Formation

Beyond the methods for binding particles, shaping them into specific geometries and pore architectures is critical. In regenerative medicine, a scaffold must conform to the defect site. While granular materials can be packed into cavities, pre-formed scaffolds that match the defect’s shape or can be easily contoured to it offer significant advantages. The optimal geometry is tissue-dependent: flat films or membranes are suitable for epithelial layers, volumetric constructs are essential for connective tissue, and tubular structures are required for nerve and muscle guidance.

Furthermore, a successful scaffold requires a macroporous structure with high interconnectivity. Pores must be at least 100 µm to facilitate cell penetration, viability, and proliferation. Pore morphology is also material-dependent; for instance, fibrous materials typically exhibit elongated pores, while foams tend to have a more spherical shape of pores [118,119].

Methods for fabricating scaffolds with controlled shapes and architectures can be broadly classified into two categories: physicochemical methods and instrumental methods.

**Figure 7:**
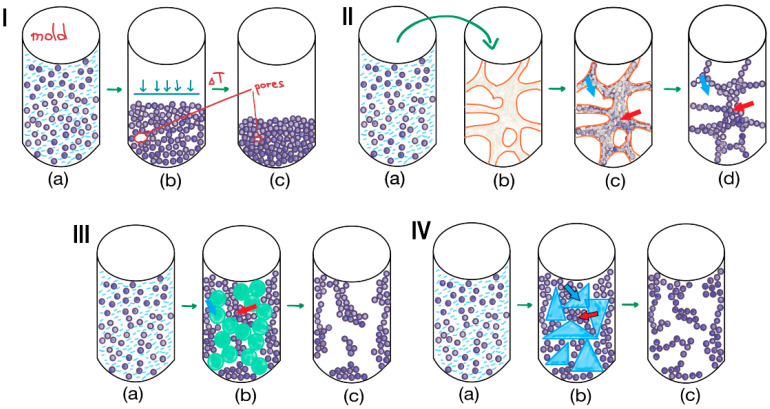
Methods for 3D structuring and pore formation: (**I**)—molding: (a)—placing a particles suspension into the mold, (b)—compression of particles, (c)—molded particles-based material; (**II**)—external templating: (a,b)—placing a particles suspension into the polyurethane sponge; (с)—removal of excess suspension from large pores of polyurethane sponge; (d)—material formed from sintered or fused particles; red arrow shows how particles aggregates form walls; blue arrow shows supermaropore of sponge and then of particles-based matrix; (**III**)—internal templating: (a,b)—emulsification of particles suspension, (b,c)—solidification of particles in the continuous phase; red arrow shows particles pushed together by emulsion; blue arrow shows emulsion drops, which allow to form supermacropores; (**IV**)—cryogelation: (a,b)—freezing of the particles suspension, (b,c)—lyophilization of the frozen solvent; red arrow shows unfrozen microphase, in which particles are pushed together by frozen solvent; blue arrow shows crystalline frozen solvent phase, which allow to form supermacropores.

**Figure 8:**
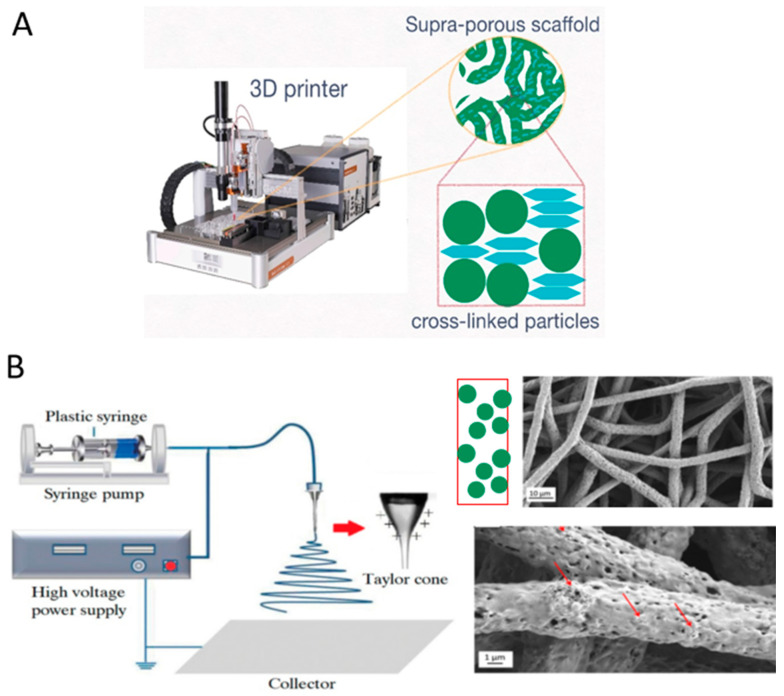
Instrumental methods for the fabrication of particle-based or particle-rich 3D scaffolds: (**A**) 3D printing based on direct ink writing combined with stereolithography (UV-mediated cross-linking of rigid (spherical elements) and soft (flattened elements) nanoparticles). Reproduced from [114] under the terms of the Creative Commons CC BY license; (**B**) electrospinning. The red arrows indicate HA particles. Adapted from [120,121]. Reproduced under the terms of the Creative Commons CC BY li-cense.

- Physicochemical methods, such as molding, templating, and cryogelation, rely on material properties and spontaneous processes to define morphology and pore structure (Figure 7).
- Instrumental methods, including 3D printing and electrospinning (Figure 8), use specialized equipment to provide the scaffold’s architectural features.

However, while some techniques enable simultaneous control over both macroscopic shape and micro-scale pore structure, others primarily control only one of these parameters.

#### 3.2.1. Molding

Molding is the manufacturing process of shaping materials into desired forms. This transformation is achieved by introducing a raw material into a mold—a hollow matrix that determines the shape and size of the final product (Figure 7I(a–c)). However, this technique typically offers control over the macroscopic shape but not the internal micro-architecture.

This type of manufacturing was successfully used for the preparation of scaffolds made of polyester nanoparticles [76]. For that, particle suspensions were just placed into the mold and gelled. Such systems formed more or less stable 3D porous materials (Figure 6), in which nanoparticles were linked together into micrometer-scale ringlike structures. However, the diameter of the pores was less than 10 µm and could not be considered sufficient for effective cell ingrowth.

The molding process is a straightforward method for achieving a desired macroscopic geometry. However, it must be combined with another technique to control pore size. Therefore, for effective scaffold fabrication, molding is often integrated with methods like sintering, templating, and cryogelation, which are discussed in the following subsections.

#### 3.2.2. Templating

Various templates can be applied to form scaffolds of diverse geometry. In contrast to molding, templating can allow control not only of material shape, but also of its porosity. Based on this, templating techniques can be classified as either external templating, which controls both the overall material shape and its internal pore structure, or internal templating, which is used specifically to engineer the porous architecture.

##### External Templating Approaches

As an example of an external templating approach, the application of highly porous polyurethane (PU) foam with interconnected, open, large pores can be considered [122]. This foam was used as a template to prepare an HA-based scaffold for bone tissue engineering. In this process, HA nanoparticles (HA NPs) were suspended in a poly(vinyl alcohol) (PVA) aqueous solution (Figure 7II(a)), and the suspension was infused into the PU sponge (Figure 7II(b,c)). The resulting matrix was subsequently dried and sintered at 1100 °C, yielding a highly porous scaffold with pore diameters of 400–600 µm (Figure 7II(d)). This “replication method” produces a porous structure that closely resembles the architecture of the foam template [123]. The technique fundamentally involves the penetration of a particle suspension into the pores of the template, followed by the destruction of the template and sintering of the particles. While excellent porosity can be achieved, this method is primarily suitable for inorganic particles like HA rather than polymeric ones.

##### Internal Templating Approaches

For assembling organic particles into porous matrices with controlled porosity, emulsion templating is particularly useful. The technique generally involves two main steps: (1) the preparation of an emulsion composed of at least two immiscible liquids, where the internal phase is dispersed in the continuous phase (Figure 7III(a,b)), and (2) the solidification of the continuous phase of the emulsion [124]. The droplets of the dispersed phase act as pore templates, which are removed after solidification to create a porous matrix (Figure 7III(c)). These biphasic emulsion systems can be either water-in-oil (*w/o*) or oil-in-water (*o/w*) depending on the polymer in the continuous phase. The solidification of the continuous phase often occurs due to polymerization. When the volume of the dispersed phase is very high, the method is called high internal phase emulsion (HIPE) polymerization and results in a highly porous polyHIPE foam [125].

A critical aspect of this approach is the stabilization of the high internal phase content, which enables the production of microcellular structures with large pore sizes (up to 1 mm). Interestingly, such an emulsion can be stabilized as a Pickering emulsion using solid particles as surfactants [126]. In these systems, pore size can be adjusted by varying the particle concentration. The stabilizing particles concentrate at the pore interfaces and can significantly influence the final scaffold’s properties.

Recently, Yuan et al. used gelatin microspheres, obtained by water-in-oil emulsion, as an internal template for a porous scaffold [127]. The scaffold was prepared according to the following procedure. First, gelatin droplets were formed in an oil phase. These droplets were then solidified into gelatin microspheres using genipin, a low-molecular-weight crosslinking agent. Second, a macromolecular crosslinker, dialdehyde amylose, was used to cluster the microspheres together through covalent cross-linking. Finally, the material was washed to remove unreacted gelatin from the microsphere interiors. Critically, the pore size was directly governed by the size of the gelatin microspheres. Although the final scaffold was not particulate in nature, the size and spatial organization of the microspheres were undoubtedly crucial in determining its overall architecture.

Recent advancements, such as a rapidly curable, biocompatible polyHIPE foam [128] have enabled the incorporation of porous, growth-factor-loaded microspheres into its structure [129]. This progress indicates that rapid in situ porous polymerization of the continuous phase is emerging as a key fabrication strategy. This is further supported by work on functionalized microspheres for injectable scaffolds [130], and the development of fast-curing emulsions using click chemistry [131].

Templating is an effective technique for proof-of-concept studies, where achieving a highly specific and ordered macro-architecture is the main goal. It offers effective control over pore size and shape, compared to random packing methods. However, due to its complexity, harsh post-processing, and inability to directly create cell-laden scaffolds, it may not be suitable for many advanced tissue engineering applications that require bioactivity, scalability, and the creation of complex hierarchical structures. For these applications, other methods, such as cryogelation, electrospinning, and 3D bioprinting, may be preferred, as they provide a more direct and biocompatible route to creating functional scaffolds.

#### 3.2.3. Cryogelation

Cryogelation is a process that induces the gelation of a particle suspension under frozen conditions. In this method, the solvent (usually water) freezes (Figure 7IV(a,b)), forming a network of ice crystals that acts as a porogen. These growing crystals exclude and concentrate dissolved polymers or suspended particles into the interstitial, unfrozen liquid micro-phase between them (Figure 7IV(b)). Subsequent sublimation (freeze-drying) or thawing removes the ice crystals, yielding a highly porous, monolithic network (Figure 7IV(c)). This technique can be applied to suspensions of pre-formed particles, e.g., polymer microspheres, ceramic granules, cellulose nanocrystals, etc., to create supermacroporous sponge-like architecture with large, interconnected pores (typically 10–200 µm). The particles are not just physically packed together. During freezing, they are concentrated in the unfrozen liquid micro-phase between ice crystals. If the particles possess surface functionality or a small amount of binder polymer is present, they become permanently fused at their contact points upon solvent sublimation. This process transforms loose particles into a robust, monolithic structure. The interparticle bonding responsible for this fusion can be either covalent cross-linking or physical interactions.

One of the first and most classical studies in this area described a one-step method for the cryostructuration of soft particles [132]. Pre-synthesized microgel particles (e.g., PNIPAAm) were dispersed in an aqueous medium at a specified concentration (typically 5 wt%). The particle suspension was cooled in an ice bath, and a chemical cross-linking agent was then added and homogeneously distributed throughout the cooled suspension. The mixture was transferred to molds and subjected to controlled freezing (e.g., −12 °C) for a prolonged period (often overnight) to facilitate cross-linking within the unfrozen micro-phase. Finally, the samples were thawed and washed to remove any residual cross-linker and unintegrated particles.

The versatility of cryogelation is demonstrated by its application to various materials. For instance, magnetically responsive gelatin cryogels have been fabricated from a suspension containing 4 wt% gelatin and 5 wt% magnetite (Fe_3_O_4_) nanoparticles. In that case, the alkaline co-precipitation of Fe^2+^ and Fe^3+^ salts with NaOH yielded magnetite crystals with an average core diameter of 10 nm. Immediately after precipitation, the particles were coated with a terpolymer shell composed of PEG, styrene, and *N*-(3-dimethylaminopropyl)-methacrylamide (DMAPM), which increased the hydrodynamic radius to approximately 200 nm and provided amine-rich surface groups for subsequent coupling. Prior to gel formation, both the functionalized particles and gelatin were pre-activated with NHS/EDC chemistry. The cryogelation protocol began by thoroughly dispersing the magnetic nanoparticle suspension into the gelatin solution in 0.1 M acetic acid at room temperature; the mixture was held for 2 h to ensure complete infiltration of the polymer shell by gelatin chains. The sol was then directionally frozen in a two-step regime (−20 °C followed by −80 °C) and freeze-dried. The resulting macroporous monoliths were further cross-linked and washed to yield the final scaffold.

In a more recent example, cryogelation was used to organize methacrylate-functionalized PLA nanoparticles and methacrylate-functionalized cellulose nanocrystals into supermacroporous materials [114]. Surface modification enabled the particles to undergo free-radical cross-linking in the unfrozen micro-phase, initiated by ammonium persulfate (APS) and accelerated by tetramethylethylenediamine (TMEDA). The precooled suspension of modified nanoparticles and reagents was mixed, transferred to a mold, and frozen at −13 °C for 24 h. The resulting cryogel was then thawed and thoroughly washed.

Another significant study demonstrated the cryostructuring of silica particle–silk fibroin composites without a covalent cross-linker [133]. In this work, mesoporous silica particles were dispersed in deionized water using sonication to achieve a homogeneous suspension. The resulting suspension was then mixed with a silk fibroin solution to form a stable hybrid. This mixture was rapidly frozen in liquid nitrogen and subsequently held at −20 °C to allow for controlled ice crystal growth, which defined the macroporous architecture. To induce physical cross-linking of the silk fibroin, the frozen construct was treated with cold methanol at −20 °C for 24 h, facilitating a conformational transition to β-sheet structures. The scaffold was then thawed at room temperature, allowing the ice crystals to melt and reveal the interconnected pore network, and finally washed to remove residual methanol.

When considering cryogelation for fabricating particle-based scaffolds, it is crucial to recognize that the final pore architecture is dictated by the morphology of the ice crystals, which depends on several key parameters: freezing rate, freezing direction, particle concentration, and size. Slower freezing rates generally produce larger ice crystals and consequently larger pores, while unidirectional freezing creates aligned, channel-like pores [134]. Notably, the overall shape of the cryogel is determined by the mold in which it is frozen. Thus, it is excellent for creating simple blocks, cylinders, or sheets, but it cannot easily create complex, predefined internal architectures.

#### 3.2.4. Electrospinning and Electrospraying

Beyond self-assembly and templating, instrumental techniques like electrospinning and electrospraying offer powerful routes for creating particle-rich scaffolds. Electrospinning is widely used to fabricate nanofibrous scaffolds that mimic the native ECM [135]. A significant advancement in this field has been the incorporation of functional particles into these fibers, creating multifunctional biomaterials that combine structural benefits with enhanced bioactive properties (Figure 8) [136].

The electrospinning process involves several critical parameters, such as voltage (typically 10–30 kV), flow rate (0.1–2.0 mL/h), and collector distance (10–25 cm), that directly influence fiber formation and particle incorporation [137]. These parameters must be optimized for each polymer–particle system to achieve uniform fiber formation and prevent particle aggregation.

Recent studies have demonstrated the successful incorporation of cerium oxide nanoparticles (CeO_2_-NPs) for their antioxidant and anti-inflammatory properties [138], zinc oxide nanoparticles (ZnO-NPs) for osteogenic applications [139], and titanium dioxide nanoparticles (TiO_2_-NPs) for wound healing [138].

Beyond nanoparticles, researchers have incorporated microparticles and composite particles. For instance, hyaluronic acid–chitosan (HA-CS) nanoparticles have been successfully electrospun with poly(ε-caprolactone) (PCL) and gelatin fibers [140]. Silica nanoparticles associated with DNA have been used for gene delivery applications [141]. Superparamagnetic iron oxide nanoparticles (SPIONs) have been incorporated into collagen scaffolds for magnetic stimulation applications [142].

Advanced electrospinning methodologies enable the fabrication of complex scaffolds. Coaxial electrospinning has been employed to create core–shell nanofibers with controlled particle distribution [143]. For example, Zhao et al. designed a new biomimetic bone scaffold using an electrospun PCL/collagen/HA shell and a core of freeze-dried collagen with icariin-loaded chitosan microspheres [144]. This drug-loaded 3D scaffold supports excellent cell attachment in vitro and has promoted abundant new bone formation in in vivo studies. Other techniques, such as multi-needle electrospinning systems, allow for the creation of 3D scaffolds with enhanced structural complexity [135]. Blend electrospinning involves the direct mixing of particles with polymer solutions before spinning, while surface modification techniques allow for post-spinning particle attachment [140].

It is important to note that in all these examples, the particles are incorporated alongside fiber-forming polymers, which provide the structural integrity for the scaffold. Electrospinning itself is not designed to create scaffolds from discrete particles alone. Methods such as particulate leaching or sintering are more suitable for fabricating purely particle-based scaffolds.

Besides electrospinning, electrospraying can also be applied to form particle-rich scaffolds [145]. Electrospray deposition is a solution-based, high-voltage atomization process that converts a dilute polymer or polymer-nonsolvent mixture into a cloud of charged micro- and nano-droplets. Because the liquid viscosity is below the threshold for fiber formation, the jet breaks up into droplets that solidify into particles or hollow microcapsules. The final morphology is governed by formulation and operating conditions. Specifically, a lower polymer concentration or the addition of a volatile non-solvent (e.g., ethanol in chloroform) promotes phase separation and yields particles with nanofibrous surfaces. Elevated humidity enhances vapor-induced phase separation, increasing surface porosity. Higher flow rates produce larger droplets and thus larger particles. The charged particles can be steered electrostatically to accumulate into predefined 2D and 3D architectures. Use of an electrostatic lens further concentrates the particle stream, enabling the direct writing of high-aspect-ratio pillars, continuous lines, and custom micro-architectures without masks or post-processing. The resulting constructs possess multiscale porosity—nanopores in the particle shell, hollow interiors, and inter-particle voids, while the fibrous outer layer offers cell-adhesive topography. Continuous production, solvent evaporation at room temperature, and the ability to switch inks on the fly confer scalability and compositional versatility. Consequently, electrospray deposition provides a straightforward route to particle-rich scaffolds for tissue engineering, drug-delivery microcarriers, porous membranes, functional coatings, and other advanced material systems that benefit from controlled morphology, high surface area, and spatially resolved assembly.

Sequential electrospinning and electrospraying methods have been developed to create bilayer scaffolds, where one layer contains the base polymer and the second layer incorporates particles [146]. This approach allows for controlled release profiles and enhanced functionality.

Although electrospinning and electrospraying are highly versatile techniques, they present distinct challenges, especially when the goal is to create scaffolds from pre-formed particles. The first is incompatibility with aqueous particle suspensions. In fact, many functional particles, such as microgels, are synthesized and stored in aqueous buffers. However, electrospinning typically requires polymer solutions in volatile organic solvents to facilitate fiber formation. The second challenge is the limited control over 3D scaffold architecture, since these techniques commonly produce scaffolds that are inherently dense and sheet-like. The third challenge in creating a scaffold by particle electrospinning or electrospraying is that they typically land and form a loose powder or very weak aggregate held together by weak forces. So, without a binding matrix or a post-processing sintering step, the scaffold lacks structural integrity and falls apart easily.

#### 3.2.5. 3D-Printing

3D-printing or additive manufacturing (AM) has revolutionized tissue engineering by enabling the fabrication of customized scaffolds with precise and complex architectures. A key advantage of AM is its ability to create patient-specific implants based on individual medical image data, offering a tailored fit for the defect site. While many 3D-printed scaffolds are fabricated from polymers, hydrogels, or composites, there is growing interest in particle-based scaffolds constructed solely from micro- or nanoparticles. These structures offer unique benefits, including high surface area, tunable porosity, and enhanced bioactivity [147,148].

The ability of particles to encapsulate various molecules and even cells makes them particularly attractive for 3D printing. Early studies described that using cell-laden microspheres in bioprinting could reduce the required initial cell density while improving the compressive strength of the construct [149,150]. Subsequent studies have described the use of microspheres for the local delivery of drugs such as small molecules [151] and growth factors [152,153]. Particles have also been used as structural support [154], and as sacrificial particles to create interconnected porous networks [155].

Several 3D-printing methods are specially designed for the fabrication of scaffolds from particles: binder jetting, selective laser sintering (SLS)/selective laser melting (SLM), electrophoretic deposition-assisted 3D-printing, and direct ink writing combined with stereolithography. In binder jetting, a liquid binding agent is selectively deposited onto a thin layer of powder, binding the particles together in a layer-by-layer fashion (Figure 8). The types of particles that can be used in this process include ceramics (HA, TCP) [156] and polymers [157]. Binder jetting does not require the application of any support, offers high resolution, and is relatively easily scalable. Processing parameters such as binder concentration, surfactant addition, and layer thickness directly affect printability, pore definition, and the mechanical and biological performance. A significant challenge for this technique, however, is achieving strong interlayer adhesion, and its print resolution is typically in the range of 20–100 µm [158].

SLS and SLM use a laser selectively to sinter or melt powdered particles, fusing them into solid structures [158]. These techniques are compatible with thermoplastics (PLLA, PCL) [159], metals, and ceramic particles [160], and can produce scaffolds with robust, strong mechanical properties without the need for binders. A significant disadvantage of this method is the use of high temperatures, especially the instant heat from the laser, which limits the number of biocompatible materials that can be used and makes the incorporation of temperature-sensitive bioactive molecules (e.g., growth factors, drugs) impossible [161]. Furthermore, SLS/SLM typically works better with particle sizes of around 100 µm.

Electrophoretic deposition-assisted 3D-printing uses electric fields to direct charged particles onto a substrate in a layer-by-layer manner [162]. For example, nanocrystalline HA and bioactive glass particles were used to form materials with this process. This approach allows fine control over particle deposition without thermal degradation. All printing operations occur at room temperature, so temperature-sensitive additives such as growth factors or drugs could, in principle, be incorporated without degradation. A significant advantage of this method over other techniques is that it can utilize very small ceramic [163] or polymer [164] particles. However, this method is primarily useful for creating thin-film coatings and gradient structures rather than large, bulky implants. Limitations include the need for a specialized membrane support and issues with reproducibility. In solvents with high permittivity like water, strong electric field gradients can cause fluid turbulence, disturbing particle deposition and leading to poor resolution and undefined shapes.

In addition to the classical methods discussed above, particles can also be organized into macroporous matrices with the application of conventional extrusion and direct ink writing methods. Recently, Klar et al. employed an extrusion-based additive manufacturing platform to implement the “3D-McMap” protocol, in which the printable feedstock was a highly concentrated suspension of monodisperse microspheres [165]. The bio-ink consisted of 200 ± 10 µm PLA or PLGA microparticles dispersed in a 3% aqueous carboxymethyl-cellulose solution. During printing, the syringe barrel was cooled to 4 °C to retard drying, whereas the build stage was maintained at 50–60 °C to accelerate solvent removal from the deposited filaments.

Deposition was interrupted after every two layers for 10 s to allow partial drying. An aluminum foil served as a sacrificial substrate, and extrusion pressure was manually adjusted to maintain a steady flow and the completed constructs were exposed to dichloromethane vapor to sinter the particle contacts and consolidate the scaffold. This approach yields scaffolds composed almost entirely of microspheres, endowing the printed filaments with intrinsic microporosity and providing a high local loading capacity for bioactive agents; it also permits spatially resolved placement of multiple particle populations and, after vapor sintering, affords compressive strengths of more than twice those of its unsintered counterparts. However, the current implementation of this method is still labor-intensive and sensitive to both environmental and process variables. The sticky, particle-rich ink demands continual operator intervention for pressure adjustment, nozzle cleaning, and layer-pause timing. It is also prone to clogging and cannot be deposited as isolated dots or segments without “stringing”.

The outstanding feature of 3D printing is the ability to exercise spatial control over the distribution of mechanical properties and biological signals within a fabricated structure. The connective tissues often possess various gradients, which should be maintained by scaffolds. In this context, particle-based systems have been demonstrated to be highly useful building blocks for constructing such graded architectures [166]. For example, Singh et al. produced uniform PLGA microspheres using the precision particle fabrication technique and either left them blank or loaded them with the model dyes rhodamine B or fluorescein to mimic distinct bioactive cues. For scaffold construction, two separate suspensions of these microspheres were placed into syringes mounted on programmable syringe pumps and delivered into a cylindrical glass mold. By imposing user-defined flow-rate profiles on each pump, the relative proportion of the two microsphere types entering the mold could be varied in real time. This process enabled the fabrication of scaffolds with bilayer, multilayer, or continuously smooth graded architectures. Once the desired packing profile had been established, the settled microspheres were soaked in ethanol for 50 min. Ethanol induced surface plasticization of the PLGA, creating a thin interconnecting film that fused neighboring particles into a continuous, highly porous matrix. The construct was finally freeze-dried to remove residual solvent and stabilize the three-dimensional scaffold.

**Figure 9:**
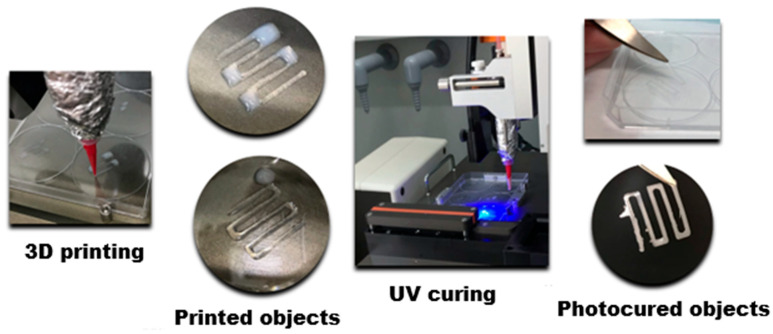
Scheme for the production of scaffolds from a mixture of methacrylated polymer nanoparticles via 3D-printing based on a combination of direct ink writing and stereolithography.

The above-mentioned protocol could be realized via a hybrid 3D printing technique combining direct ink writing (DIW) and stereolithography (SLA) [114]. For example, Leonovich et al. used an aqueous suspension of methacrylated PLA and CNC particles (PLA-MA and CNC-MA) as “ink” [114]. The suspension was also supplemented with a macromolecular cross-linker, GelMA, which acted as a “molecular glue”. Its addition significantly enhanced the formation of a stable 3D network between the particles during UV-curing. For printing, the particle-based bioink was loaded into a syringe and extruded through a fine nozzle (0.254 mm diameter) onto a platform (Figure 9). This step deposited the material layer-by-layer according to a pre-designed CAD model, defining the scaffold’s macro-architecture. After each layer was deposited, the printed layer was immediately irradiated by a UV light (365 nm). This UV exposure triggered a free-radical polymerization that cross-linked the particles and GelMA via their methacrylate groups. This in situ curing solidified the layer, fixing the particles in place and providing the scaffold with mechanical integrity. The discussed study employed a single syringe extruder for the particle mixture (PLA-MA and CNC-MA) [114]. However, using two separate syringes, each containing a different particle type, is a route for creating gradient scaffolds.

Granular hydrogels are extremely interesting materials within the context of scaffold fabrication via 3D printing. Microgels exhibit excellent shear-thinning behavior that makes them ideal for extrusion-based 3D printing. A key advantage of cross-linked microgel systems is their ability to create self-supporting structures immediately after printing. It was demonstrated that the reversible dynamic covalent cross-links in granular hydrogels provide self-healing properties that confer stability after extrusion without any additional cross-linking [102]. This eliminates the need for post-printing curing steps that are often required with many bioinks. Authors successfully printed grids, hexagons, and lines and reported that the geometric accuracy of the printed structures was slightly improved when using 1 mm extrusion tips relative to 0.4 mm. The printed structures maintained their shape when a granular hydrogel was printed as a 5 mm cylinder tube with 50 layers. This tube demonstrated elasticity and mechanical stability without post-printing cross-linking.

Another study presented an advanced triple-click chemistry approach for 3D printing. The authors applied gelatin–norbornene–carbohydrazide microgels that could be dynamically stiffened and annealed them into granular hydrogels through three orthogonal reactions. This allows for precise control over mechanical properties both during and after printing [117].

Despite their promise, several challenges persist in the 3D printing of particle-based scaffolds. A primary issue is particle agglomeration within the bio-ink, which can clog nozzles and affect printability. Another significant issue is weak interparticle bonding; unbound particles are prone to collapse. To overcome this, a post-printing consolidation step, such as thermal sintering, solvent vapor fusion, or chemical cross-linking, is frequently necessary to ensure structural integrity.

Despite these limitations, 3D-printed particulate scaffolds offer several promising perspectives for future advancement. A key perspective is multi-material printing, which enables the spatial integration of different functional particles, for instance, combining growth factor-releasing microspheres with structurally reinforcing granules. Another frontier is in situ bioprinting, which involves depositing scaffolds directly into anatomical defects using bioactive particles. The development of advanced stimuli-responsive particles for direct ink writing, which can solidify or modify their properties upon exposure to physiological cues (e.g., temperature, pH, or light), is particularly promising for realizing these advanced applications [58,167].

## 4. Properties of Particle-Based Scaffolds

### 4.1. Mechanical Properties and Morphology

Particle-based scaffolds show considerable promise for tissue engineering applications due to their capacity to form unique structures, create gradients, and deliver bioactive agents. However, their practical utility is governed by critical mechanical and morphological properties. A primary challenge is the balance between mechanical strength and the high porosity required for nutrient diffusion, vascularization, and cell migration. Furthermore, the particle surface texture directly influences cellular behavior; while irregular surfaces can enhance cell attachment, they may also provoke undesirable inflammatory responses. Finally, a uniform particle distribution is crucial for consistent scaffold performance and avoiding points of weakness.

Mechanical properties of scaffolds are very important for their performance and have been addressed by many researchers. This section first reviews literature in which the introduction of particles helped to tune the mechanics of the scaffolds. For instance, Chowdhury et al. investigated porous ceramic scaffolds fabricated from TCP, HA, and a mixed HA-TCP (50:50 wt%) composite using the polyurethane sponge replication method [168]. This technique produced particle-based scaffolds, whose architecture was a direct 3D replica of the original sponge, yielding a highly interconnected macroporous network with pore sizes exceeding 250 µm. Nanoindentation tests (to 600 nm depth) revealed that the composite HA + TCP scaffolds exhibited significantly superior mechanical properties compared to those made of pure TCP. The Young’s modulus of the composite was 10.3 GPa versus 1.5 GPa of pure TCP. Similarly, the hardness values of the composite scaffold were 240 MPa compared to 21 MPa for TCP. These results demonstrate that incorporating HA substantially enhances the mechanical performance of biodegradable scaffolds while preserving the interconnected porous architecture essential for bone tissue engineering.

The introduction of silica-based particulate materials, namely bioactive glass 4555, diatomaceous earth, and biosilica into marine-derived collagen materials has improved the compressive modulus of scaffolds. The scaffolds with incorporated particles also exhibited decreased water uptake compared to pure collagen scaffolds, leading to more stable and cohesive structures [37].

A recent study demonstrated that adding nano-CaP particles to AP40mod glass–ceramic powder fundamentally alters the scaffold properties [71]. The nanoparticles inhibit densification during sintering, increasing porosity (70% vs. 60%) and creating a finer, smoother surface morphology compared to the pure, coarse AP40mod. The nanoparticles also promote the β-TCP-to-HA transformation, enhancing the scaffold’s bioactivity. However, nanopowder content exceeding 10 wt% impairs flowability and printability, requiring higher binder saturation (110%) and limiting processability. These findings confirm that even small amounts of nano-CaP significantly modify sinterability, microstructure, and printing behavior, enabling the use of bioactive calcium phosphate in binder jetting, albeit with a compromise between enhanced porosity and manufacturability.

Ceramic nanoparticles are also well-known for enhancing the mechanical properties of polymer scaffolds. For instance, a study on electrospun PLA/HA composite scaffolds demonstrated this effect clearly [169]. The incorporation of HA significantly increased the elastic modulus, with nanoHA providing superior reinforcement—a 140–170% increase—compared to a 70% increase with microHA. The effect on tensile strength, however, depended on fiber alignment: it decreased by 68–70% in random mats but increased in aligned mats by ~130% with µHA and ~84% with nHA. All composites showed increased brittleness, with elongation at break reduced by 70–85%. Morphologically, the scaffolds formed porous nanofibrous networks. The aligned fibers were 10–20% thinner and more uniform than random fibers. NanoHA produced smoother fibers with well-dispersed small agglomerates (<4 µm), while microHA formed large irregular agglomerates (5–20 µm) that created stress concentration points, reducing reinforcement efficiency. Fiber alignment amplified HA’s benefits, with aligned composites maintaining strength while achieving higher stiffness. The optimal formulation, a PLA containing 20% nHA, combined maximum stiffness with a favorable morphology for bone tissue engineering.

However, it should be considered that the introduction of particles can very often result in the formation of fragile structures. For example, in PLGA/bioactive glass composite scaffolds, increasing the bioactive glass content from 20 to 50% led to high porosity (73.7–76.1%) up to 200 μm but also caused severe mechanical fragility that precluded quantitative testing [78]. This particle-based composition directly dictated the scaffold’s properties: higher bioactive glass content disrupted polymer continuity, creating irregular particles that reduced sintering quality and caused mechanical fragility.

In contrast to the composite scaffolds discussed above, scaffolds constructed solely from particles represent a significant area of investigation and are worthy of discussion. Morphology and mechanical properties for some selected scaffolds obtained from particles of different compositions via different approaches are summarized in Table 2. Notable examples in this category are colloidal gels, which can form structures with considerable mechanical stability. For instance, scaffolds formed from aggregated chitosan particles demonstrated robust mechanical properties, with a wet-state storage modulus of 4.2 MPa and a dry-state compressive modulus of 132 MPa [170]. These scaffolds exhibited predominantly elastic behavior with a significant damping capability (tan δ > 0.08), making them suitable for dissipating cyclic mechanical loads. Morphologically, they displayed 27.78% porosity with exceptionally high interconnectivity of 95%. They featured an average pore diameter of 265 μm and a heterogeneous pore size distribution spanning the optimal 100–400 μm range for tissue ingrowth. This combination, achieved through natural interparticle spacing, resulted in a trabecular bone-like architecture. The particle-based composition proved critical to this outcome. The average particle size of 430 μm directly determined the pore dimensions, while chitosan’s bioadhesive properties enabled strong interparticle bonding at contact points during drying. This, in turn, eliminated the need for additional binders and achieved an optimal balance between mechanical strength and interconnected porosity without complex processing or templates.

The properties of such colloidal systems are highly tunable. A study on ionically crosslinked dextran microsphere scaffolds demonstrated that the shear modulus (G’) could be precisely adjusted from ~30 Pa to 6500 Pa by varying the solid content from 12.5 to 25% [74]. These scaffolds demonstrated robust elastic behavior with low energy loss (tan δ~0.06) and nearly complete recovery after deformation. Morphologically, the structure consisted of packed microspheres (7.5–8.3 µm) whose interstitial spaces formed a macroporous network. A critical solid content of ≥12.5% was necessary to form a percolating network through extensive ionic crosslinks. Furthermore, the gel’s stability was environmentally sensitive, as low pH or high ionic strength could dissolve the structure back into a dispersion by disrupting the electrostatic interactions.

Colloid gels can also involve hard and soft particles, which result in better control over their mechanical properties. The following study employed hybrid colloid gels of soft gelatin nanoparticles and hard particles (bioactive glass, silica) to create uniform, interconnected networks through diffusion-limited cluster aggregation [70]. This composite architecture leads to synergistic mechanical improvements: incorporating bioactive glass doubled the storage modulus, while gelatin–silica composites achieved storage moduli over 50 kPa, which corresponded to a tenfold increase over pure gelatin gels. These composites exhibited robust linear–elastic behavior under both compression (modulus: ~94 kPa, strength: ~44 kPa) and tension (modulus: ~41 kPa, strength: ~8.4 kPa), confirming that the strategic combination of hard and soft particles can produce structurally stable and mechanically superior scaffolds.

**Figure 10:**
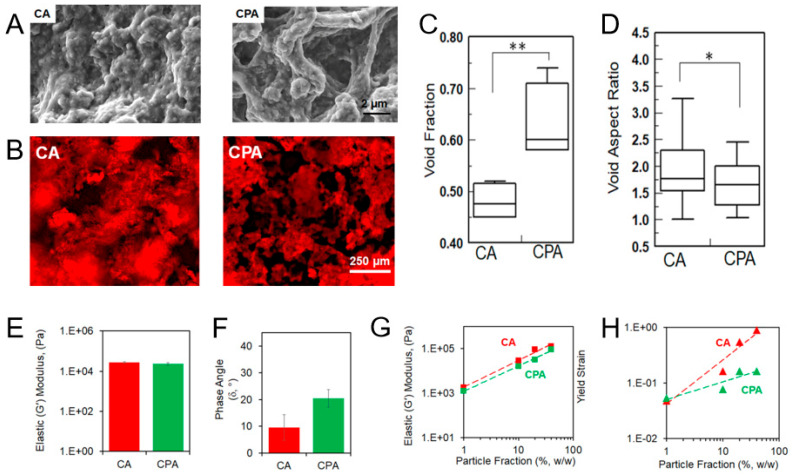
Microstructure and mechanical properties of materials based on colloidal aggregation of cationic PU particles (∼0.5 μm). CA—materials formed by aggregation of particles in PBS; CPA—materials formed by poly(acrylic acid) mediated aggregation. (**A**) Microstructured morphology of CA and CPA gels from scanning electron microscopic images; (**B**) confocal scanning fluorescent images of CA and CPA gels; (**C**) void fraction and (**D**) void aspect ratio of the materials analyzed by measuring the corresponding parameters from confocal scanning fluorescent images by using ImageJ 1.52. * The difference was not statistically significant. ** The difference was statistically significant; (**E**,**F**) elastic moduli (G′) and phase angle (δ) of CA and CPA materials obtained from the linear viscoelastic region of the strain amplitude sweep measured at constant frequency; (**G**,**H**) variation in elastic moduli (G′) and yield strain of CA and CPA gels prepared from different particle fractions; measured from the linear viscoelastic region and from the cross-over point of elastic (G′) and viscous (G′′) moduli. Adapted and reproduced with permission from [105], Copyright© 2019, American Chemical Society.

The outstanding study by Yuan et al. showed the possibility of controlling the architecture of particle-based scaffolds [105]. The authors aimed to apply the possibilities of colloidal gels to create distinct and well-controlled matrices capable of organizing endothelial cells into specific vascular structures. The scaffolds consisted of cationic polyurethane colloidal particles with spherical morphology and submicrometer dimensions of approximately 570 nm. They were synthesized from polycaprolactone-based polyurethane and featured positively charged quaternary amine groups. Two distinct scaffold architectures were created through controlled aggregation: (1) compact aggregates (CA) formed with phosphate-buffered saline exhibited 48% porosity with constricted, localized void spaces, and (2) compact polyelectrolyte aggregates (CPA) formed with poly(acrylic acid) displayed a higher porosity of 63%, with interconnected void networks and more circular pore geometry. Despite their different microstructures, both scaffold types demonstrated similar elastic moduli of approximately 25 kPa, indicating that bulk mechanical stiffness was independent of internal architecture. However, the CA scaffolds exhibited more solid-like behavior with lower phase angles compared to CPA scaffolds, reflecting their denser compact structure. The transformation from a fluidic particle dispersion (elastic modulus ~1.5 Pa) to a solid gel structure following aggregation was confirmed by frequency-independent gel-like mechanical responses. Furthermore, the CPA scaffolds’ interconnected networks enabled flow upon yielding at lower strains, whereas the denser CA scaffolds showed greater yield strain at higher particle fractions (Figure 10). The promising biological properties of these scaffolds will be discussed in a subsequent section.

The cryogelation methods described above can yield structures with highly advantageous properties. For instance, cryogels fabricated from gelatin and magnetic nanoparticles formed highly anisotropic, interconnected pore networks (50–300 µm diameter) with high porosity (~81–88%) [173]. The magnetic (M-Gel) variant exhibited superior fluid uptake, swelling to 657% compared to 564% for pure gelatin cryogels. Over 28 days, M-Gel cryogels degraded by ~40% indicating a controllable resorption profile. Although the mechanical properties and injectability of these scaffolds were not characterized, their straightforward two-step synthesis produced covalently cross-linked and magnetically responsive scaffolds. This combination of high swelling capacity, tailored biodegradation, and remote magnetic actuation makes them highly promising for advanced biomedical applications.

Interestingly, quite small nanoparticles can be formed through cryogelation into nanoparticle-based aerogel scaffolds [171]. These can form self-supporting, monolithic superstructures with ultra-low density (20–60 mg cm^−3^) and exceptional porosity (99.4–99.7%). Their delicate, filigree nature requires freeze-drying to prevent collapse from capillary forces during processing. Morphologically, they consist of a non-ordered interconnected network of thin sheets or platelets (10–100 nm thick) formed via the two-dimensional assembly of primary nanoparticles (3.5–120 nm). This creates a hierarchical pore structure with mesopores within sheets and macropores between them. The properties of the scaffold are critically dependent on particle concentration. A critical threshold of ~0.1% volume fraction is essential to form a continuous network, with higher concentrations yielding thicker sheets. Below this threshold, the structure collapses during processing. The assembly mechanism is universal across different material types (metals, metal oxides) as mechanical integrity stems from a physical, freezing-driven process where nanoparticles are expelled into ice crystal boundaries, not specific chemical bonds. This results in a consistent sheet-like morphology that preserves the nanoscale properties of the primary building blocks.

**Figure 11:**
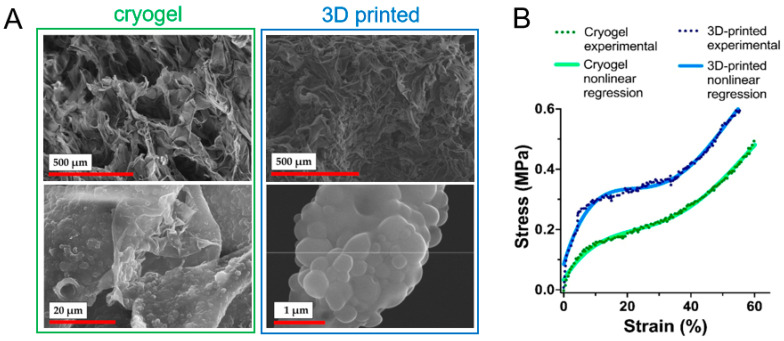
Comparison of morphological and mechanical properties of particle-based cross-linked cryogels and 3D-printed matrices: (**A**) Scanning electron microscopy images for matrices obtained by cryogelation/3D printing of methacrylated PLA nanoparticles and methacrylated CNC with gelatine-methacrylate as cross-linker; (**B**) Strain–stress test curves for cryogeleted and 3D-printed matrices. The compression stress was applied with an initial force of 0.1 N. The speed of compression was 2 mm/min. Reproduced from [114] under the terms of the Creative Commons CC BY license.

Particle-based scaffolds composed of methacrylated PLA and nanocrystalline cellulose exhibit highly composition-dependent mechanical properties. The storage modulus increases dramatically from 30 to 40 Pa for particles alone to ~2000 Pa with a PEGDA cross-linker and ~4000 Pa with a multifunctional GelMA cross-linker. Furthermore, 3D-printed scaffolds demonstrate superior compressive strength compared to softer hydrogel-like cryogelated scaffolds as a result of their denser architecture [114]. Morphologically, the two fabrication techniques produce distinct structures. Cryogelated scaffolds display supermacroporous structures with interconnected pores, averaging around 200 µm diameter, formed through ice-templating. In contrast, 3D-printed scaffolds feature a precisely controlled architecture dictated by CAD models and characterized by rough strand surfaces and smaller pores (10–100 µm). Critically, both methods successfully incorporate PLA-MA and CNC-MA particles into the pore walls of the three-dimensional network. The particle-based composition significantly influences scaffold properties, as the optimal 80:20 PLA-MA: CNC-MA ratio provides mechanical reinforcement through a synergistic combination of soft spherical and rigid needle-like particles (Figure 11). Furthermore, the cross-linker selection is pivotal. The multifunctional GelMA creates denser, more mechanically robust networks than bifunctional PEGDA and, crucially, enhances cell adhesion threefold due to its bioactive RGD peptides, despite the scaffold’s lower surface area. The fabrication method serves as the primary determinant of macro-scale architecture, with cryogelation producing isotropic, randomly interconnected pores ideal for cell migration, while 3D printing enables precise structural control and superior mechanical robustness through its layer-by-layer material deposition.

Quite recent research on the application of GelMA particles as an ink for 3D printing clearly showed that the UV-initiated free-radical photo-crosslinking process dramatically enhances mechanical strength, with storage modulus and compressive Young’s modulus increasing more than tenfold, while transitioning the material from a viscoelastic colloid gel to a purely elastic solid. The final 3D matrix becomes permanently stable and covalently locked, making it resistant to dissolution or breakdown under physiological conditions [93].

The studies on granular hydrogels have shown that jammed microgel-based structures are very weak materials with storage moduli ranging from 0.5 to 8.0 kPa. In such structures, the mechanical strength depends on the individual microgel properties, including stiffness and contact area, which are controlled by packing density, microgel size, and stiffness interactions that affect deformation patterns [174]. Annealed granular hydrogels become significantly stronger through chemical treatment, achieving storage moduli between 0.15 and 39.00 kPa, which rival solid bulk hydrogels, because the strong chemical bonds formed during annealing overcome the weakening effects of porosity [52,102]. The specific annealing chemistry employed critically determines the final stiffness, with methods like the inverse electron-demand Diels–Alder reaction producing stiffer scaffolds than thiol-ene click chemistry due to secondary interactions such as π-π stacking [172]. Thus, annealing fundamentally transforms loosely packed, mechanically weak jammed structures into strong, cohesive materials with mechanical properties comparable to non-porous bulk hydrogels through the formation of permanent inter-microgel bonds.

As mentioned above, a crucial and promising feature of particle-based scaffolds is their inherent ability to form functional gradients. Recently, it was shown that HA particles (HAP) undergo distinct morphological changes across the interface depth [175]. Newly formed HAP were flakelike, irregular crystals, which then aggregated into spheres of approximately 100 nm in diameter at shallow positions. With increasing depth, the volume of the HAP further increased, and the shape became irregular at depth. This complex assembly of nanoscale structures and components enhanced energy dissipation at the osteochondral interface, achieving a smooth stress transition between soft and hard tissues. The gradient assembly of HAP can effectively dissipate the joint stresses to avoid stress concentration at the interface region. Thus, this research provides a basis for the construction of complex interfacial regions and demonstrates how a change in composition and structural gradient assembly enables the design of biological composite interface materials.

#### 4.1.1. Comparison of Conventional and Particle-Based Scaffolds

The fundamental difference between conventional bulk and particle-based scaffolds lies in their structural paradigm: conventional scaffolds comprise a continuous solid phase with an embedded, interconnected pore network. In contrast, particle-based scaffolds are granular assemblies of discrete, usually spherical units. This foundational distinction gives rise to significant differences in their fabrication, properties, and functional applications [52].

The mechanical behavior of conventional scaffolds is that of a continuous solid. Properties like tensile strength and elongation at break are defined by the bulk material’s composition and crosslink density (Table 3). Particle-based scaffolds exhibit unique yield-stress fluid and solid-like behaviors. Furthermore, the mechanical properties of particle-based scaffolds are highly tunable and modular. The stiffness of the final construct depends on both the stiffness of the individual microgels and their jamming/annealing density.

Thus, conventional scaffolds provide a continuous, stable 3D environment that excels in applications requiring strong structural integrity and direct cell–matrix interactions throughout the entire volume. However, their dense nanoscale mesh can limit cell proliferation and infiltration. In contrast, the granular gel structure is highly conducive to cell invasion and proliferation. Cells can readily migrate through the inter-particle voids [117]. Microgel scaffolds offer unparalleled control over porosity, mechanics, and biological functionality. Their injectability, printability, and the ability to be dynamically tuned post-fabrication make them a more versatile and biomimetic platform for a wide range of applications in regenerative medicine, 3D bioprinting, and advanced in vitro disease modeling. The choice between the two ultimately depends on the specific application’s requirements for structural integrity versus dynamic cellular interactions.

#### 4.1.2. Degradability of Particle-Based Scaffolds

The degradation of particle-based scaffolds can significantly differ from that of conventional bulk scaffolds. The mechanism of particle-based scaffold degradation can be surface erosion or bulk erosion, depending on the particle size, intra-particle crosslinking, and material. A high surface-area-to-volume ratio accelerates degradation, so smaller microgels degrade faster.

The degradation of sintered scaffolds is typically a slow, surface-driven process, contrasting sharply with the bulk degradation of many polymers. For bio-ceramics like sintered hydroxyapatite, degradation occurs primarily through passive dissolution in the physiological environment, which can take months to years. In polymer-based sintered scaffolds, hydrolysis of the polymer chains is the main mechanism, but the high crystallinity and dense particle fusion from sintering significantly retard this process. This controlled, slow degradation profile is ideal for applications like bone tissue engineering, as it provides long-term mechanical support while gradually transferring load to the newly forming tissue [181,182].

The degradability of particle-based scaffolds is fundamentally controlled by three interconnected factors: the composition of the constituent particles, the cross-linking density within individual particles, and the secondary annealing strategy used to assemble them [52]. Naturally derived biopolymers such as gelatin and hyaluronic acid possess inherent enzymatic degradability through specific proteases like collagenase and hyaluronidase, whereas synthetic polymers like PEG require deliberate modification with enzyme-sensitive peptide sequences to achieve bio-responsiveness. The degradation rate exhibits an inverse relationship with cross-linking density, as tightly cross-linked networks with higher mechanical stiffness degrade more slowly than their softer counterparts.

Beyond material composition, the choice of annealing chemistry significantly impacts scaffold stability, with certain cross-linking methods like inverse electron demand Diels-Alder (iEDDA) click chemistry conferring greater resistance to enzymatic degradation compared to thiol-ene approaches [172]. The degradation process actively modulates cellular behavior by creating spatial opportunities for cell migration and expansion, with faster degradation enabling mesenchymal stem cells and endothelial cells to form complex tissue-like networks. As scaffolds degrade and soften mechanically, they can trigger beneficial cellular responses including enhanced extracellular matrix secretion and favorable shifts in macrophage polarization from pro-inflammatory to anti-inflammatory phenotypes [183]. The temporal degradation profile serves as a critical mechanism for controlling the localized and sustained release of therapeutic cargo, including drugs, proteins, and genetic materials [102]. Consequently, the degradation characteristics of particle-based scaffolds must be precisely tailored to match their intended applications, balancing the need for rapid host cell integration in regenerative medicine with the requirement for stable microenvironments in controlled in vitro studies.

Thus, particle-based scaffolds offer modularity and tunable degradation, ideal for drug delivery, injectable therapies, and rapidly remodeling tissues.

### 4.2. Biological Properties of Particle-Based Scaffolds

#### 4.2.1. Enhancements in Biological Characteristics When Incorporating Particles

It is known that particles have an interesting effect on the biological properties of scaffolds even when they are not structural components of the scaffold (Table 4). In particular, it was shown in several studies that the introduction of functional particles into scaffolds profoundly enhances their biological properties through multiple interconnected mechanisms. These particles fundamentally transform the cell–material interface by modifying surface chemistry and topography, as demonstrated by ECM fragments providing ligand-rich surfaces that improve cell attachment efficiency [184], and alginate residues converting hydrophobic PLA surfaces into more bioactive substrates that promote cell spreading [185]. Furthermore, the use of alginate as a porogen generates interconnected pore networks and micropores, which enhance nutrient diffusion and enable deeper cell infiltration throughout the 3D construct [185].

One of the interesting advantages of granular hydrogels is their ability to incorporate cells and provide them with a complex microenvironment [52]. Two primary strategies exist for incorporating cells into granular hydrogel scaffolds: (1) seeding cells onto microgel surfaces and interstitial spaces after scaffold formation; (2) encapsulating cells within microgels during fabrication. While the first method offers simplicity and avoids exposing cells to potentially toxic fabrication processes, the latter one protects cells from shear forces during extrusion, enhances nutrient diffusion through high surface-area-to-volume ratios, and provides interstitial expansion space [116,186]. Both strategies have proven successful across diverse regenerative medicine applications [180,186], with surface seeding being favored for straightforward applications and encapsulation preferred when bioprinting protection, enhanced nutrient exchange, or cryopreservation capabilities are required. Granular hydrogel scaffolds loaded with cells have been successfully applied to cartilage tissue regeneration, new blood vessel formation, and skin wound healing [187,188].

**Table 4:** Effect of particle nature and characteristics on biological properties and performance of scaffolds.

Scaffold and Particles	Study Design	Cells/Tissue	Observed Effects	Ref.
I. Scaffolds with incorporated particles				
Laponite nanoclay + PEGDA/Alginate/Gelatin	In vitro	Mouse embryonic fibroblasts (NIH 3T3)	Increased metabolic activity of cells	[189]
Chitosan + Chitosan NPs and ZnO NPs	In vitro and In vivo	Baby hamster kidney fibroblast cells (BHK-21); Subdermal tissue of male Wistar rats	Facilitated the formation of HA	[190]
Silk fibroin and mesoporous silica particles	In vitro	Mouse fibroblast cells (L929)	Cell attachment and growth within the scaffold’s 3D structure	[133]
Collagen-HA composite scaffolds	In vitro	THP-1, MSCs	HA particle shape and size showed great influence on osteoimmunomodulatory cues	[191]
II. Scaffolds made solely of particles				
Colloid gel: Gelatin NPs, bioactive glass NPs	In vitro and In vivo	MC3T3 pre-osteoblast cell line; Femoral condyle bone tissue in an osteoporotic rat model	Enhanced biomineralization; Localized antiosteoporotic effect	[101]
PU colloidal particles	In vitro	HUVEC cultured in endothelial cell growth medium without VEGF	Endothelial cells interconnected with stranded colloidal networks and formed capillary-like structures with enhanced cell–matrix interactions and increased cell extension; Cells were elongated and interconnected longitudinally to form network-like structures.	[105]
Colloidal gel: Gelatin NPs and calcium phosphate nanospheres	In vitro	Bone marrow stem cells (BMSCs)	Support for attachment, spreading, and proliferation of BMSCs; Advantageous cell attachment due to RGD sequences in gelatin.	[75]
PLLA NPs	In vitro and In vivo	MCF-7 and B16 cells; Full-thickness skin wounds on the dorsum of rats	Showed faster wound closure compared to the control group.	[56]
Biphasic calcium phosphate nanopowder derived from cuttlebones mixed with glass-ceramic powder (sintering).	In vitro	Human mesenchymal stem cells (hMSCs)	Scaffolds promoted fast cell adhesion and proliferation; Metabolic activity decreased over time for gradient pore scaffolds with cuttlebone-derived powder, possibly indicating cell differentiation; Cells effectively colonized the porous structure.	[71]
Porous composite microspheres made of PLGA and bioactive glass BG 45S5	In vivo	Bone cells (osteoblasts), fibroblasts, cells involved in neovascularization, and cells for skin regeneration	Bioactive glass shows superior bone regeneration and dissolution rates compared to HA; Bioactive glass in PLGA composites buffers pH during degradation, tailoring degradation behavior; Open porosity and varied pore sizes are crucial for cell nutrition, proliferation, and migration; Different pore sizes support the regeneration of different tissues.	[78]
Oppositely charged PLGA NPs: PLGA-chitosan and PLGA-alginate (injectable scaffolds)	in vitro	Human umbilical cord mesenchymal stem cells (hUCMSCs)	Viability tests of hUCMSCs seeded on the colloidal gels demonstrated negligible cytotoxicity of the materials	[92]
Methacrylated PLA and methacrylated CNC + methacrylated gelatin as cross-linker	In vitro	Bone marrow mesenchymal stem cells (MSCs) and mouse fibroblast cells (NIH 3T3) for cytotoxicity testing.	Modification of PLA and CNC nanoparticles by methacrylate groups did not result in a drastic increase in their toxicity; Adhesion to 3D printed material was significantly greater than that in the case of cryogels.	[114]
Hyperbranched polyethylene glycol and thiolated gelatin granular hydrogel	In vitro	Human Dermal Fibroblasts, Human Epidermal Keratinocytes	Formation of a multi-layered epidermis, complete with a keratinized stratum corneum	[69]
Carboxybetaine acrylamide, sulfobetaine methacrylate microgels crosslinked with alginate methacrylate.	In vitro	Human primary articular chondrocytes, THP-1 monocyte cell line	High chondrocyte viability, significant secretion of glycosaminoglycans and collagen, compressive modulus of the cell-laden constructs increased approximately 10-fold after 21 days of culture	[192]

#### 4.2.2. Pharmacological Properties of Particle-Based Scaffolds

Beyond surface effects, particles can serve as dynamic reservoirs of biochemical signals that directly influence cell fate. VEGF-loaded nanoparticles sustain endothelial cell proliferation and viability, while cell-derived ECM particles drive robust osteogenic differentiation and mineralization of mesenchymal progenitors [193]. Most significantly, such particle systems can orchestrate complex physiological responses. For example, VEGF-releasing particles increased in vivo blood vessel density from ~1.8 to 6.1%, thereby transforming scaffolds from passive structural supports into active instructive platforms for tissue regeneration.

The granular hydrogel scaffolds quite often incorporate various functional biological signals, primarily consisting of proteins, peptides, small molecular compounds, and genetic components [52,183,194,195]. Protein-derived biological cues can be classified into two main categories: factors promoting cell proliferation and molecules that regulate inflammatory responses. In particular, regulatory factors controlling growth and cellular differentiation—including VEGF, transforming growth factor β (TGF-β), and recombinant human bone morphogenetic protein 2 (rhBMP-2)—are most frequently utilized to promote mesenchymal stem cell expansion and direct their transformation into specific cell types necessary for tissue regeneration [196,197].

**Figure 12:**
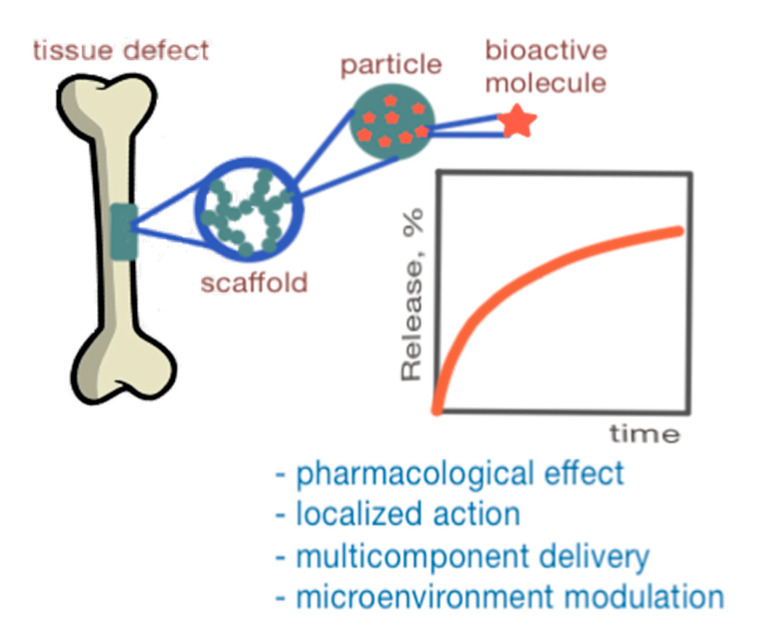
Schematic representation of the principle of a pharmacologically active scaffold for tissue engineering.

The integration of bioactive particles into scaffold systems has revolutionized the field of tissue engineering by transforming passive structural supports into pharmacologically sophisticated delivery platforms (Table 5, Figure 12). The fundamental pharmacological advantage of particle-incorporated scaffolds lies in their ability to achieve controlled and sustained release of bioactive compounds. By manipulating particle characteristics such as crosslinking density, size, and composition, release profiles can be tailored to match the specific temporal requirements of tissue healing.

Gelatin nanospheres demonstrate this principle effectively: low-crosslinked particles (GelA) enable rapid release suitable for early-phase angiogenic factors like FGF-2, while high-crosslinked particles (GelB) provide sustained release appropriate for prolonged osteogenic signaling via BMP-2 [198]. This controlled release mechanism prevents the initial burst release phenomenon common to many conventional carriers, maintaining therapeutic concentrations throughout critical healing phases while avoiding toxic peak concentrations.

Functional particle-based scaffolds enable the execution of complex, multi-phase therapeutic strategies through the sequential release of diverse bioactive substances [100]. This advanced capability is crucial for mimicking the natural temporal progression of tissue healing, where different biological signals must be presented at specific stages. The dual growth factor delivery system exemplifies this approach: early release of angiogenic factors stimulates vascularization of the defect site, establishing a blood supply network, followed by the sustained release of osteogenic factors that drive bone formation once the vascular infrastructure is established. This sequential presentation creates a coordinated regeneration cascade that recapitulates physiological bone healing more faithfully than simultaneous or single-factor approaches.

Beyond sequential delivery, particle systems enable multi-component strategies that combine complementary or synergistic agents. Scaffolds can simultaneously deliver anabolic bone-forming factors such as BMPs alongside anti-catabolic agents like alendronate that inhibit osteoclastic bone resorption, creating a balanced microenvironment that maximizes net bone formation [199]. Similarly, combining osteogenic signals with anti-inflammatory compounds like lactoferrin or ibuprofen modulates the immune response, preventing chronic inflammation that would otherwise impede regeneration. The synergistic effects observed with multi-component delivery frequently surpass the additive effects of individual agents, achieving superior tissue regeneration outcomes while maintaining lower, safer doses of each component.

**Table 5:** Pharmacological effect of application of particles with biologically active components.

Scaffold and Particles	Encapsulated Molecules	Study Design	Cells/Tissue	Observed Effects	Ref.
Porous Ti scaffolds produced by selective laser melting + nanostructured colloidal gels composed of oppositely charged gelatin nanospheres	BMP-2, FGF-2	In vivo	Osteoprogenitor cells/Cortical bone defects in rat femurs	Continuous bone formation throughout 12 weeks	[198]
Electrospun nanofiber scaffolds + PLGA nanoparticles	Gentamicin sulfate	In vitro release and antibacterial testing	No mammalian cells or tissues, Escherichia coli	antibacterial nanofiber scaffolds with controlled gentamicin release	[200]
Biomimetic Calcium Phosphate (BioCaP) particles	BMP-2	In vitro and in vivo	Osteoblasts and progenitor cells/ Rat cranial bone tissue	Significantly more new bone formation compared to just biphasic calcium phosphate, and comparable to autologous bone	[201]
PLLA + PLGA nanoparticles	VEGF	In vitro and ex vivo	MC3T3-E1, HUVECs/ chick chorioallantoic membrane	increased collagen production by osteoblasts, significant vasculature development in the chorioallantoic membrane	[202]
Electrospun PCL + decellularized ECM nanoparticles	Insoluble and soluble bioactive molecules of ECM	In vitro	Human adipose-derived stem cells (hASCs)	Osteogenesis of hASCs differentially promoted or impeded depending on the tissue of origin of the ECM	[203]
Electrospun nanofiber scaffolds + PLGA nanoparticles	Gentamicin sulfate	In vitro Release and antibacterial testing	No mammalian cells or tissues, Escherichia coli	Antibacterial nanofiber scaffolds with controlled gentamicin release	[200]
Hyaluronic acid methacrylate and sulfated hyaluronic acid methacrylate microgels	Transforming Growth Factor-β3 and Platelet-Derived Growth Factor-BB	In vitro Cartilage defect model	Human Bone Marrow-derived Stromal Cells	Much greater cell migration and infiltration compared to bulk hydrogels	[204]

The pharmacological functionalization of scaffolds profoundly influences cellular behavior at multiple levels, beginning with the enhanced recruitment of regenerative cells to the defect site. Released chemotactic factors, such as BMPs, PDGF, and various peptides, act as potent attractants for mesenchymal stem cells and osteoprogenitor cells, concentrating these critical cell populations within the scaffold architecture. Upon arrival, cells encounter a carefully orchestrated biochemical environment where released bioactive molecules engage specific cellular receptors and activate intracellular signaling pathways, including BMP, Wnt, and Hedgehog cascades that drive osteogenic differentiation [199].

A critical pharmacological advantage of particle-incorporated scaffolds is their capacity for localized drug delivery directly at the target site. This spatial confinement maximizes therapeutic efficacy at the defect while minimizing systemic exposure and potential off-target effects. For antibiotic-loaded scaffolds, such as those containing gentamicin-encapsulated PLGA nanoparticles [200], localized delivery achieves high antimicrobial concentrations at the wound site without systemic toxicity, which is particularly important for aminoglycosides, which are known for nephrotoxicity and ototoxicity with systemic administration. The uniform distribution of nanoparticles throughout the scaffold structure ensures consistent drug availability across the entire defect area, preventing undertreated zones that could harbor infection or fail to regenerate.

In summary, the evolution of scaffold systems built of bioactive particles marks a significant advancement in regenerative medicine. They transcend the role of structural templates to become active platforms that provide precise spatiotemporal control over the regenerative microenvironment. This capability to deliver multi-factorial therapy locally and sustainably makes them a uniquely powerful tool for addressing complex reconstructive challenges and advancing regenerative outcomes in even the most challenging clinical scenarios.

#### 4.2.3. Types of Tissues and Effects

**Figure 13:**
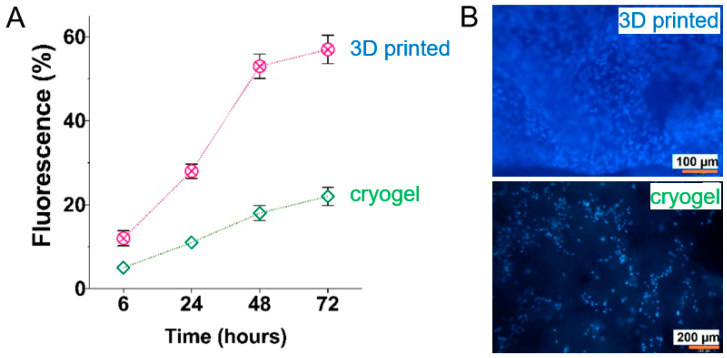
Proliferation of MSCs on the surface of cross-linked particle-based matrices fabricated via cryogelation or 3D-printing using methacrylated PLA and methacrylated CNC, and cross-linker poly(ethylene glycol) dimethacrylate for cryogelation or gelatin–methacrylate for 3D-printing: (**A**) Fluorescence analysis of DAPI-stained MSCs at 6, 24, 48, and 72 h post-seeding. (**B**) Representative fluorescence images of DAPI-stained MSCs at 72 h. Reproduced from [114] under the terms of the Creative Commons CC BY license.

A majority of studies have reported enhanced bone formation or osteogenic differentiation with particle-based scaffolds. In studies on bone repair, such scaffolds stimulate osteogenesis by increasing collagen production, alkaline phosphatase activity, and mineral deposition, while also promoting angiogenic responses such as endothelial tubule formation and VEGF upregulation. Czekanska et al. reported increased collagen production by osteoblasts and stimulation of progenitor cells with PLGA nanoparticle-decorated scaffolds [202]. One study observed a synergistic effect on osteogenesis and angiogenesis when bioactive glass nanoparticles were incorporated into a nanofibrous matrix [205]. Another study found that cobalt-doped particles enhanced both osteoblast differentiation and vascular gene expression [206]. Mousa et al. provided a comprehensive review of the literature on the use of clay nanoparticles for enhancing osteogenic differentiation and mineralization [207]. Leonovich et al. showed that the introduction of gelatine into particle-based scaffolds significantly enhances stem cell attachment and spreading, thus setting a trend for connective tissue formation [114] (Figure 13).

A minority of studies, such as Angarita et al.’s work, focused on soft tissue but still reported particle-based scaffold-induced matrix organization and cell viability [190]. Some studies targeting bone regeneration reported angiogenic effects. For instance, Czekanska et al. demonstrated that VEGF release from PLGA nanoparticle-decorated scaffolds significantly enhanced vasculature, as evidenced by increased endothelial cell tubule formation and robust vascular development in a chorioallantoic membrane assay [202]. Quinlan et al. showed that cobalt ions released from bioactive glass particles upregulated VEGF expression in human umbilical vein endothelial cells (HUVECs) and promoted tubule formation [206]. Kim et al. reported enhanced migration and tubule networking of HUVECs on bioactive glass nanoparticle/nanofibrous scaffolds with in vivo evidence of neovascularization [205].

**Figure 14:**
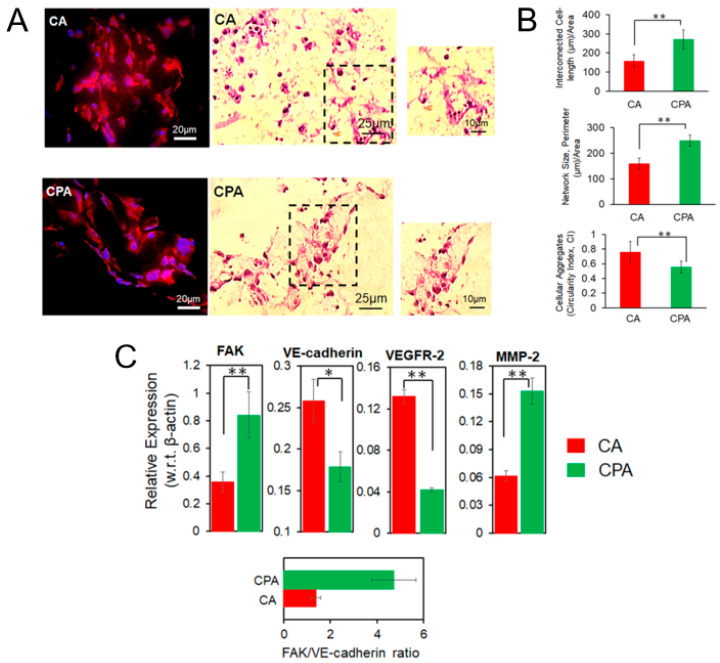
Effect of particle organization on biological properties of the scaffold. CA—materials formed by aggregation of particles in PBS; CPA—materials formed by poly(acrylic acid)-mediated aggregation. (**A**) Endothelial cell (EC) organization and morphogenesis in colloidal gels. Representative fluorescent images (red = actin and blue = nucleus) and H&E stained images of ECs in CA and CPA colloidal gels; (**B**) Morphometric analysis of EC organization measured by analyzing the total length of interconnected cell chord per unit area, size (perimeter) of enclosed network form EC chords, and shape of cellular aggregates in CA and CPA materials; (**C**) EC signaling: levels of focal adhesion kinase (FAK), VE-cadherin, VEGFR2, and MMP2 in ECs measured by immunoblot analysis with β-actin as loading control in CA and CPA materials. Statistics: ANOVA, ** *p* ≤ 0.01, * *p* ≤ 0.05. Adapted and reproduced with permission from [105], Copyright© 2019, American Chemical Society.

One of the fundamental challenges in the regeneration of complex tissues is understanding how the microstructural morphology of a three-dimensional matrix provides spatial reference points for the organization of endothelial cells into specific vascular structures. Yuan, etc., conducted in vitro experiments using HUVECs cultured in endothelial cell growth medium without VEGF in colloidal aggregate-embedded matrices and in colloidal gels [105]. The scaffold microarchitecture dramatically influenced endothelial cell organization patterns (Figure 14). Scaffolds with compact dense architecture promoted compact cellular aggregates with enhanced cell–cell interactions and lumen-like structure formation within constricted voids, while scaffolds with open loose microstructure facilitated elongated capillary-like networks with enhanced cell–matrix interactions and longitudinal interconnections. Molecular signaling analysis revealed that open, loose microstructured scaffolds upregulated focal adhesion kinase and matrix metalloproteinase-2 expression, indicating enhanced cell–matrix adhesion and matrix remodeling capacity. At the same time, compact dense scaffolds showed elevated VE-cadherin and VEGFR-2 expression, reflecting strengthened cell–cell adherent junctions and receptor signaling. Morphometric quantification demonstrated that the relative performance of compact, dense scaffolds versus open, loose ones depended on the surrounding matrix composition: the former generated greater tube network length and junctions in Matrigel systems, while the latter produced superior tubulogenesis in collagen-based systems. Cell viability remained high across all scaffold formulations with no significant differences compared to cell-only controls, and endothelial cells exhibited longer sprouting distances in loose microstructure matrices regardless of the surrounding matrix type. Time-dependent analysis over 48 h revealed progressive increases in endothelial organization features in both scaffold types, confirming their capacity to support dynamic vascular network development.

Thus, the reviewed studies reported that particle-based scaffolds were associated with both osteogenesis and angiogenesis in laboratory and animal models. There are also some studies on injectable particle-based scaffolds for soft tissue regeneration [92].

## 5. Future Outlook

Particle-based scaffolds represent a powerful tool in regenerative medicine, offering versatility and control over scaffold properties through the tailored assembly of micro- and nanoparticles as primary building blocks. This review has examined how particles of diverse composition, ranging from bioceramics like HA and TCP to synthetic polymers such as PLGA, and natural biopolymers like gelatin and chitosan, can be organized into 3D architectures. Various mechanisms include sintering, electrostatic aggregation, and covalent cross-linking, which enable precise architectural control and provide the means to create scaffolds for regenerative medicine. The resulting constructs offer remarkable mechanical tunability, ranging from soft, injectable gels to rigid, load-bearing structures. They also achieve high porosity and interconnectivity, which are essential for cell infiltration, nutrient transport, and vascularization.

The modular nature of particle-based scaffolds provides unique advantages that distinguish them from conventional monolithic materials. By precisely selecting and combining particles with different sizes, compositions, surface modifications, and functional properties, researchers can create heterogeneous structures that more faithfully replicate the complexity of native ECM. The ability to incorporate multiple particle populations enables the creation of spatial gradients in mechanical stiffness, biological signals, and pharmacological agents—features that are challenging to achieve with traditional scaffold fabrication approaches. Furthermore, the intrinsic capacity of particles to encapsulate and controllably release bioactive molecules transforms these scaffolds from passive structural supports into active therapeutic platforms capable of tissue regeneration through sequential delivery of growth factors, cytokines, and other signaling molecules.

### 5.1. Innovations in Methods for Scaffold Production

The convergence of particle-based scaffold design with cutting-edge additive manufacturing technologies represents one of the most promising frontiers for future development. While current 3D printing approaches have demonstrated proof-of-concept for particle-based constructs, significant opportunities exist for refinement and innovation. Next-generation bioprinting systems could employ multiple print heads containing different particle suspensions, enabling real-time fabrication of scaffolds with precisely controlled spatial distributions of mechanical properties, degradation rates, and biological cues. The integration of machine learning algorithms could optimize print parameters dynamically during fabrication, adjusting for particle size distribution, viscosity changes, and environmental conditions to ensure consistent quality and reproducibility.

Hybrid manufacturing approaches combining 3D printing with other techniques such as electrospinning or electrospraying could create hierarchical structures with particle-based bulk regions providing mechanical integrity and fibrous surface layers optimizing cell attachment. The development of stimuli-responsive particle systems that undergo shape changes, property modifications, or controlled release in response to temperature, pH, enzymatic activity, or external fields (magnetic, electric, ultrasound) would enable the creation of “smart” scaffolds capable of adapting to the evolving microenvironment during tissue regeneration. Such 4D-printed particle scaffolds could expand to fill irregular defects, stiffen in response to mechanical loading, or release therapeutic agents triggered by inflammatory signals.

### 5.2. Multifunctional Particle-Based Scaffolds

Future particle-based scaffolds will likely incorporate increasingly complex multifunctional particles designed to address multiple aspects of tissue regeneration simultaneously. Core–shell particles with distinct interior and exterior compositions could combine mechanical reinforcement with surface bioactivity, while multi-compartment particles could enable independent control over the release kinetics of multiple therapeutic agents. The integration of sensing capabilities within particles, such as fluorescent reporters of pH, oxygen tension, or specific enzymes, would enable real-time monitoring of the regeneration process and provide feedback for adaptive therapeutic interventions.

Theranostic particle systems combining therapeutic and diagnostic functions represent a particularly exciting area. Particles incorporating contrast agents for medical imaging modalities (MRI, CT, ultrasound) alongside growth factors or stem cells would allow non-invasive tracking of scaffold degradation, cell migration, and tissue formation throughout the regeneration process. This would facilitate personalized medicine approaches where treatment protocols could be adjusted based on individual patient responses observed through longitudinal imaging studies

### 5.3. Biomimetic and Bioinspired Designs

Advancing our understanding of natural tissue architecture and ECM organization will inform the next generation of particle-based scaffolds with enhanced biomimetic properties. Particles designed to mimic the size, shape, and surface properties of natural collagen fibrils, proteoglycans, or other ECM components could create synthetic microenvironments more closely resembling native tissues. The strategic combination of particles presenting different cell-adhesive ligands, matrix-binding domains, or protease-sensitive sequences could recapitulate the biochemical complexity and dynamic remodeling capacity of natural ECM.

Bio-inspired approaches might also draw from non-mammalian systems such as nacre, whose brick-and-mortar architecture provides exceptional mechanical properties through the organized layering of inorganic platelets and organic matrix. Translating such principles to particle-based scaffolds through controlled particle shape, orientation, and interfacial chemistry could yield materials with superior strength, toughness, and damage tolerance for load-bearing applications.

### 5.4. The Need to Expand Tissue Applications

While much research has focused on bone regeneration due to the natural affinity between hard inorganic particles and osseous tissue, significant opportunities exist for expanding particle-based approaches to other tissue types. Soft tissues such as cardiac muscle, neural tissue, and vascularized organs present unique challenges requiring softer, more compliant particles with specialized functionalities. Hydrogel-based particles, lipid particles, or polymer particles with carefully tuned degradation kinetics matched to the slow regeneration rates of neural tissue could address these needs.

Interfacial tissues such as osteochondral regions, tendon–bone insertions, and myotendinous junctions, characterized by gradients in composition, structure, and mechanical properties, represent ideal applications for multi-particle scaffolds. By creating smooth or stepwise transitions in particle composition, size, and organization, scaffolds could better replicate these complex interfaces and potentially reduce failure rates at tissue boundaries that plague current approaches.

### 5.5. Manufacturing and Translational Challenges

Realizing the full clinical potential of particle-based scaffolds requires overcoming several manufacturing and regulatory challenges. Scalable, reproducible production methods ensuring consistent particle properties (size distribution, surface chemistry, drug loading) across batches will be essential for regulatory approval and commercial viability. Development of quality control standards specifically for particle-based scaffolds, including metrics for particle distribution homogeneity, interparticle connectivity, and release profile consistency, will facilitate translation from laboratory to clinic.

Addressing the economic considerations of particle-based scaffold production through process optimization, automation, and economies of scale will be crucial for accessibility. While custom, patient-specific scaffolds may justify premium pricing for high-value applications, developing standardized particle libraries and modular assembly protocols could reduce costs for broader applications. Partnerships between academia, industry, and regulatory bodies will be essential for establishing manufacturing guidelines, sterilization protocols, and clinical testing frameworks appropriate for these novel materials.

## 6. Concluding Remarks

Particle-based scaffolds have evolved from a niche research area into a mature and versatile platform technology with transformative potential for regenerative medicine. The capacity to precisely engineer scaffold properties across multiple hierarchical length scales—from nanoscale surface features and particle composition to microscale interparticle interactions and macroscale architectural organization—provides unprecedented design flexibility that cannot be achieved with conventional bulk hydrogels or traditional scaffold fabrication methods. This multi-scale control enables the creation of biomimetic microenvironments that more faithfully recapitulate the spatial, mechanical, and biochemical complexity of native tissues.

The field has now reached a critical juncture where fundamental scientific advances must be strategically translated into clinically viable solutions. Several key priorities emerge from this review. First, standardization of particle synthesis and scaffold fabrication protocols is essential to ensure reproducibility across laboratories and facilitate meaningful comparison of results. Second, the development of predictive computational models that link particle properties, assembly strategies, and biological outcomes would accelerate rational scaffold design and reduce empirical optimization. Third, systematic investigation of scaffold degradation kinetics and their correlation with tissue remodeling dynamics remains critically needed to optimize temporal matching between scaffold dissolution and neo-tissue formation. Fourth, integration of advanced biofabrication technologies—such as bioprinting, microfluidics, and programmable assembly—with particle-based systems represents a frontier for achieving patient-specific, anatomically complex tissue constructs.

Looking forward, particle-based scaffolds are uniquely positioned to address several of the most challenging needs in regenerative medicine: engineering vascularized tissues of clinically relevant dimensions, creating biomimetic interfaces for osteochondral defects, developing immunomodulatory matrices for challenging wound healing environments, and establishing tumor-mimetic platforms for precision oncology research. The fundamental advantages inherent to particle-based approaches—modularity enabling compositional heterogeneity, tunability across multiple property dimensions, multifunctionality through orthogonal particle populations, and biomimetic potential through bottom-up assembly—position them not merely as an incremental improvement but as a paradigm shift in how we conceptualize and construct tissue engineering scaffolds. As interdisciplinary collaborations deepen and manufacturing technologies mature, particle-based scaffolds stand ready to serve as a cornerstone platform for next-generation regenerative therapies that can restore form and function to damaged or diseased tissues.

We believe that this review will facilitate research in the area of particle-based scaffolds by providing a comprehensive overview of current approaches, identifying key challenges and opportunities, and inspiring new directions for investigation. The continued development and translation of particle-based scaffolds promise to transform the treatment of tissue damage and loss, ultimately improving outcomes and quality of life for millions of patients worldwide facing injuries, diseases, and age-related tissue degeneration.

## Figures and Tables

**Table 1:** Types of particles and methods in particle-based scaffold formation.

Chemical Composition	Hard/Soft	Size	Type of Interparticle Interaction	Type of Material and Fabrication Method	Ref
Gelatin, silica	Soft Hard	400 nm 80 nm	Non-covalent aggregation	Colloidal gel by pH induced gelatin NPs aggregation	[70]
Natural biphasic calcium phosphate, glass-ceramic powder	Hard Hard	405 nm, 6 µm 45–100 µm	Sintering	3D-disks with separated pores; 3D-printing and sintering	[71]
HA, TCP	Hard Hard	63 nm	Sintering	3D-matrix with interconnected pores; Sol–gel process followed by polymer foam replication technique	[72]
Poly(lactide-*co*-glycolide) with incorporated HA	Semi-hard	250–500 µm (HA particles 14 nm)	Sintering	3D-matrix, composed of interconnected particles; Sintering in a pre-designed stainless-steel mold	[73]
Dextran-HEMA * copolymerized with MAA * or DMAEMA *	Soft	8.3–7.5 µm	Electrostatic interaction	Colloid gel; Mixing of particle suspensions	[74]
Gelatin, calcium phosphate nanocryslals (needles)	Soft Hard	380–480 nm Length 173 nm Width 30 nm	Electrostatic Interaction	Colloid gel; Mixing of particle suspensions	[75]
Gelatin A, Gelatin B	Soft	200 nm 150 nm	Electrostatic interaction	Colloid gel; Mixing of particle suspensions	[57]
PLGA-PVAm ** PLGA-PEMA **	Semi-hard	181 nm 144 nm	Electrostatic interaction	Colloid gel; Mixing of particle suspensions	[76]
polyamide 12 HA	Semi-hard Hard	50–80 µm 40–100 µm	Sintering	3D-printed matrices; additive manufacturing via selective laser sintering	[55]
PLA-chitosan	Semi-hard	60–70 µm	Sintering	Layers of sintered microparticles; Surface-selective laser sintering	[77]
PLGA composite microspheres with bioactive glass as porogen	Semi-hard Hard	40–50 µm 1.7 µm	Sintering	Cuboid specimens; laser sintering machine	[78]
4-arm polyethylene glycol (PEG) vinyl sulfone cross-linked by dicysteine MMP-sensitive peptide	Soft	50 μm	Covalent cross-linking	Granular hydrogel	[79]
hyperbranched polyethylene glycol and thiolated gelatin	Soft	50, 100, 150 μm	Covalent cross-linking	Granular hydrogel	[69]

*—HEMA—2-hydroxyethylmethacrylate; MAA—methacrylic acid; DMAEMA—dimethylaminoethyl methacrylate; **—PLGA-PVAm—poly(lactic acid-co-glycolic acid) modified by poly(vinylamine); PLGA-PEMA—PLGA modified by poly(ethylene-co-maleic acid).

**Table 2:** The morphology and mechanical properties of scaffolds obtained from particles of different composition via different approaches.

Particles	Processing Method	Interconnectivity and Porosity	Pore Size or Other Characteristics	Mechanical Properties	Ref.
TCP and nanosized HA	PU sponge method (template approach and sintering)	Highly interconnected (SEM)	>250 μm (SEM)	E = 10.3 GPa H = 240 MPa	[168]
Ca phosphates and glass ceramic	3D jet printing followed by sintering	Total porosity 60–70%	Pore size gradient 1170/620/340 μm	Bulk density 1.4–1.6 g/cm^3^	[71]
PLGA and bioactive glass	Laser sintering	Porosity > 73%	Pore size 200 μm	Bulk density 0.15–0.16 g/cm^3^ Low strength, brittle	[78]
Oppositely charged dextran microsphere	Colloid gel formation due to ionic association	Amount of free water (~70%) characterizes the porosity	Particle size in the wet gel ~8 μm	Shear modulus (G’) 30–6500 Pa, Viscoelastic material	[74]
Soft gelatin and hard silica nanoparticles	Colloid gel formation due to pH change	Uniform interconnected particulate networks	gelatin NPs size ~400 nm, silica NPs size ~80 nm	Compression test: E = 93.8 kPa Tensile test: E = 40.6 kPa Fracture strain: ≈23%; Self-healing after cut	[70]
Noble metal and metal oxide	Cryogelation (freeze-drying)	Porosity > 99%	N/A	Poor mechanical stability	[171]
Cationic PU colloidal particles	Electrostatic interaction-mediated aggregation	48–63 void space	Pore size from 85 to 100 μm	Storage Modulus: ≈25 kPa	[105]
PLA, microHA and nanoHA	Electrospinning, PLA as polymer solution, and HA as filler	Highly porous, porosity depends on aligning	Pore size depends on alignment, >30 μm according to SEM	Elastic modulus 3–25 MPa; Tensile strength 1–20 MPa (depending on HA concentration)	[169]
Methacrylated PLA and methacrylated CNC	Cryogelation or 3D direct ink writing followed by interparticle cross-linking	Highly porous gels	Pore size: cryogels ~200 μm 3D printed matrices ~100 μm	Storage modulus (G’) 2000–4000 Pa	[114]
Chondroitin sulfate with either methacrylate, aldehyde, or hydrazide groups	Mixing and jamming using vacuum filtration to form	Dynamically cross-linked granular hydrogels with porosity of more than 10%	~150 μm	Compressive modulus ~17 kPa Max stress ~10 kPa	[102]
Norbornene-functionalized PEG microgels	Electrospraying followed by thiol-norbornene click cross-linking	~40–47%	Pore size area ~2.3 × 10^3^ μm^2^	Storage Modulus ~1–2 kPa	[172]

**Table 3:** Comparison of the morphology and mechanical properties of scaffolds produced from bulk polymers and using particles.

Scaffold Materials	Preparation Method	Pore Size (Porosity)	Mechanical Properties	Ref.
Chitosan solution	Freeze-drying	50–250 µm (highly porous)	Young’s Modulus ~5 kPa	[176]
Chitosan spheres	Particle aggregation	40–262 µm (55%) Controlled by size of microspheres	Young’s Modulus ~53 kPa	[177]
PLA filament	3D Printing (FDM)	>500 µm (20–100%)	Tensile Strength 4–28 MPa, depends on porosity	[178]
Oppositely charged PLGA nanoparticles	Colloidal Gel Assembly	0.1–10 µm (>80%) according to SEM	Flow behavior with high viscosity	[76]
Type A Gelatin	Microfluidic 3D Foaming	100–160 µm (highly porous)	Tensile Strength: ~8.4–10.8 kPa, depends on pore size	[179]
Alginate + Gelatin Methacrylate microgels	Air-Assisted Co-Axial Jetting—microgels	50–500 µm (highly porous)	Storage Modulus: ~2–250 kPa, depends on composition + self-healing properties	[180]
Gelatin-Norbornene-Carbohydrazide microgels	Aggregation and annealing via click-chemistry	~130–230 µm (~30–40%)	Tunable Microgel Stiffness, Storage Modulus: ~1.8 kPa–10 kPa	[117]

## Data Availability

No new data were created or analyzed in this study. Data sharing is not applicable to this article.

## Abbreviations

The following abbreviations are used in this manuscript:

ECM	Extracellular matrix
CNC	Cellulose nanocrystals
PLA	Poly(lactic acid)
PDLLA	Poly(D,L-lactic acid)
PLLA	Poly(L-lactic acid)
HA	Hydroxyapatite
HAP	Hydroxyapatite particles
TCP	Tricalcium phosphate
HEMA	2-Hydroxyethylmethacrylate
DMAEMA	Dimethylaminoethyl methacrylate
PLGA	Poly(lactic acid-*co*-glycolic acid)
PVAm	Poly(vinylamine)
PEMA	Poly(ethylene-*co*-maleic acid)
SLS	Selective laser sintering
SEM	Scanning electron microscopy
Dex	Dextran
Gel	Gelatine
PNIPAM	Poly(*N*-isopropylacrylamide)
PVA	Poly(vinyl alcohol)
PU	Polyurethane
HIPE	High internal phase emulsion
NHS	*N*-hydroxysuccinimide
EDC	1-Ethyl-3-(3-dimethylaminopropyl)carbodiimide
APS	Peroxodisulfate
TMEDA	*N,N,N,N*-tetramethylethylenediamine
CS	Chitosan
PCL	ε-Caprolactone
AM	Additive manufacturing
SLM	Selective laser melting
DIW	Direct ink writing
SLA	Sterelithography
CA	Compact aggregates
CPA	Compact polyelectrolyte aggregates
VEGF	Vascular endothelial growth factor
FGF	Fibroblast growth factor
BMP	Bone morphogenetic factor
hASCs	Human adipose-derived stem cells
HUVECs	Human umbilical vein endothelial cells
MC3T3-E1	Embryonic mouse calvarial cell line
PDGF	Platelet-derived growth factor
